# Toward Understanding the Brain Dynamics of Music: Learning and Conscious Performance of Lyrics and Melodies With Variable Rhythms and Beats

**DOI:** 10.3389/fnsys.2022.766239

**Published:** 2022-04-08

**Authors:** Stephen Grossberg

**Affiliations:** Center for Adaptive Systems, Graduate Program in Cognitive and Neural Systems, Department of Mathematics & Statistics, Psychological & Brain Sciences, and Biomedical Engineering, Boston University, Boston, MA, United States

**Keywords:** music, lyrics, melody, rhythm, consciousness, musical beat, Adaptive Resonance Theory, working memory

## Abstract

A neural network architecture models how humans learn and consciously perform musical lyrics and melodies with variable rhythms and beats, using brain design principles and mechanisms that evolved earlier than human musical capabilities, and that have explained and predicted many kinds of psychological and neurobiological data. One principle is called *factorization of order and rhythm*: Working memories store sequential information in a rate-invariant and speaker-invariant way to avoid using excessive memory and to support learning of language, spatial, and motor skills. Stored invariant representations can be flexibly performed in a rate-dependent and speaker-dependent way under volitional control. A canonical working memory design stores linguistic, spatial, motoric, and musical sequences, including sequences with repeated words in lyrics, or repeated pitches in songs. Stored sequences of individual word chunks and pitch chunks are categorized through learning into *lyrics chunks* and *pitches chunks*. Pitches chunks respond selectively to stored sequences of individual pitch chunks that categorize harmonics of each pitch, thereby supporting tonal music. Bottom-up and top-down learning between working memory and chunking networks dynamically stabilizes the memory of learned music. Songs are learned by associatively linking sequences of lyrics and pitches chunks. Performance begins when list chunks read word chunk and pitch chunk sequences into working memory. Learning and performance of regular rhythms exploits cortical modulation of beats that are generated in the basal ganglia. Arbitrary performance rhythms are learned by adaptive timing circuits in the cerebellum interacting with prefrontal cortex and basal ganglia. The same network design that controls walking, running, and finger tapping also generates beats and the urge to move with a beat.

## 1. Introduction

### 1.1. Lyrics, Melodies, Rhythm, and Beat

This article proposes brain design principles, mechanisms, and architectures that enable humans to learn and consciously perform lyrics and melodies with variable rhythms and beats. There are currently a number of excellent articles and books that discuss facts about music and about how our minds perceive it (e.g., Gjerdingen, 1989; Howell et al., 1991; Deutsch, 1992, 2013; Krumhansl, 2000; Repp, 2005, 2006a,b; Levitin, 2006; Zatorre et al., 2007; Thompson, 2009; Large, 2010; Patel and Iversen, 2014; Large et al., 2015; Nguyen et al., 2018; Rajendran et al., 2018; Damm et al., 2020). The current article complements these contributions by developing a neural model of the brain mechanisms that regulate how humans consciously perceive, learn, and perform music. The article’s exposition is non-technical and illustrates its proposals with analyses of specific melodies and songs.

The article proposes how music builds upon brain mechanisms that are used in multiple perceptual, cognitive, and motor processes. It shows how variations of the same types of neural circuits that can store lyrics or melodies can be used to oscillate with a beat. These unifying mechanistic insights contribute to understanding how music may have emerged through evolution from brain processes that earlier evolved to carry out more basic psychological functions. Indeed, these variations have elsewhere been used to qualitatively explain and quantitatively simulate on the computer many kinds of psychological and neurobiological data.

### 1.2. Bach’s Partita No. 1 for Piano Illustrates That Musical Groupings Are Short

I will first introduce musical notation to illustrate that many musical groupings are short. I will then explain how short musical groupings make possible how we learn and perform music.

**FIGURE 1:**
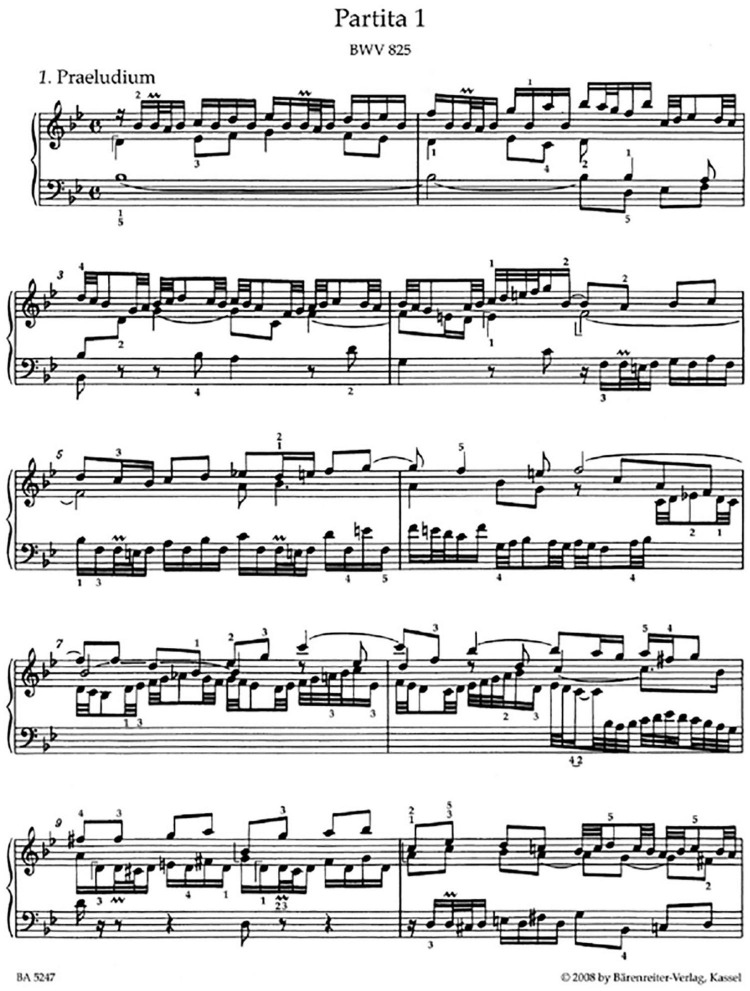
Page 1 from the score of J. S. Bach’s Partita #1 for piano, BWV 825.

Figure 1 copies the first page of Johann Sebastian Bach’s Partita No. 1. The incomparable Glenn Gould plays it here: https://www.youtube.com/watch?v=7pj5r8anMdc. Figure 1 illustrates the information that musical notation embodies. A brief review of musical notation is included to make the article accessible to those who do not read music.

Piano sheet music is organized into two separate rows of notes. The *treble clef*, also called the G clef, describes higher sounding notes, which are usually played with the right hand. The *bass clef*, also called the F clef, describes lower sounding notes, which are usually played with the left hand.

Consider the notes to the right of the treble clef in the top row of the music. They are linked, or grouped together by horizontal bars, or beams, either above or below the notes that they link. These groupings influence how the piece is practiced and encoded in long term memory.

The first horizontal bars lie above the first four notes printed in the treble clef. The next horizontal bars lie above the fifth through eighth notes. Each bar also indicates the relative speed with which these grouped notes are played. Two bars command the right hand to play these notes twice as fast as notes that are linked by one bar, such as the notes below them that are played by the left hand. Finer structure is depicted among the second and third notes of the treble clef that are linked by a triple bar and are therefore played even faster. The jagged line, or chevron, above the second note commands that note to be played even more quickly as part of a trill.

The two ♭ shaped symbols that are printed right after the clefs define the key: They require that notes b and e be played on the “flat” black keys that lie on the piano keyboard just before the white keys for b and e.

The music is further divided by vertical lines, which separate the music into bars. The C shapes to the right of the clefs and the ♭ symbols denote 4/4 time. The upper 4 means that there are four beats in a bar, and the bottom number 4 says which kind of note will receive one beat, which, in this case, is the quarter note. The first black note to be played by the left hand is a quarter note. Thus, the notes within each bar are played in four beats that are equally spaced through time.

This segment of music illustrates that groupings are often short, here four or five notes long, with more notes playable in a given amount of time at a faster speed. This fact raises basic questions, including:

• Why are musical groupings so short?
• How do musical key and harmonic relationships constrain the notes that are played in musical groupings?
• How do variable numbers of notes get fit to an underlying beat?

### 1.3. Musical Grouping: Harmonics, Pitch, Streams, Arpeggios, and Tonality

The kind of grouping that is marked in a piece of music like Bach’s Partita is influenced by several different kinds of grouping constraints that are due to the physics of sound and the properties of hearing.

The physics of sound determines one crucial source of grouping; namely, the *pitch* of a sound, or note, in music. The pitch of a sound is a psychological percept that determines how high or low the sound is consciously heard in any piece of music. The perceived pitch typically depends on the fundamental auditory frequency of the sound, with higher fundamental frequencies sounding like higher pitches. Harmonics, or frequencies that are integer multiples of the fundamental frequency, are overtones that all contribute to the percept of pitch. Thus the pitch percept is the result of another form of grouping. In particular, the harmonics that a pitch percept represents are bound together to learn a *pitch category*.

**FIGURE 2:**
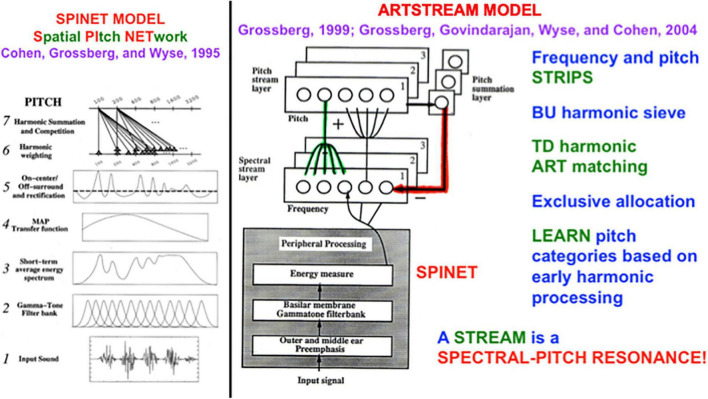
(left panel) The Spatial Pitch Network, or SPINET, model (Cohen et al., 1995) shows how a log polar spatial representation of the sound frequency spectrum can be derived from auditory signals occurring in time. This spatial representation allows the ARTSTREAM model to compute spatially distinct auditory streams. (right panel) The ARTSTREAM model explains and simulates the auditory continuity illusion as an example of a spectral-pitch resonance. Interactions of the ART Matching Rule and asymmetric competition mechanisms in cortical strip maps explain how the tone selects the consistent frequency from the noise in its own stream while separating the rest of the noise into another stream. [Reprinted with permission from Grossberg et al. (2004)].

Figure 2 (left panel) summarizes the Spatial Pitch NETwork, or SPINET, model that I developed with Michael Cohen and Lonce Wyse (Cohen et al., 1995) to explain the brain processing stages that begin with incoming sounds and end with pitch categories. The SPINET model was used to quantitatively simulate many human psychophysical data about pitch perception, including data about: the phase of mistuned components, shifted harmonics, dominance region, octave shift slopes, pitch shift slopes, pitch of narrow bands of noise, rippled noise spectra, tritone paradox, edge pitch, and distant modes. The amount of psychophysical data that the model explains and simulates assured that SPINET could be used to provide inputs to auditory streaming processes.

*Auditory streams* are another source of grouping that enables the Bach Partita, and indeed all other music, to sound like a continuous flow of sound, even though we consciously hear only discrete notes through time. Gjerdingen (1994, p. 335) has discussed the conscious perception of auditory streams during music, noting that “a great deal of the motion perceived in music is apparent rather than real. On the piano, for example, no continuous movement in frequency occurs between two sequentially sounded tones,” say in an arpeggio, “though a listener may perceive a movement from the first tone to the second.” Properties of arpeggio playing were modeled by Gjerdingen (1994) using a neural model of apparent motion in vision that was introduced and developed by Grossberg and Rudd (1989, 1992).

Apparent motion in vision occurs when a discrete series of lights that are placed in a linear row turn on sequentially through time. When the spacing of the lights, and the timing with which they are sequentially lit, are within an appropriate range, one perceives a continuous motion between them in the order in which they are lit. Analogously, apparent motion in music occurs when a discrete series of tones that are organized tonotopically in a linear row turn on sequentially through time within an appropriate range of rates. The brain mechanism that causes both percepts is the same; namely, when each light or tone is turned on, it activates a Gaussian receptive field that is centered at that light or tone. Gaussian receptive fields are ubiquitous in our brains. Successive activations, within an appropriate range of rates, of lights or tones whose Gaussian receptive fields overlap across space can cause a traveling wave of continuous activation to flow across each network from the first to the second light or tone. I call this traveling wave a *G-wave*, or Gauss-wave. Remarkably, a simple process like a G-wave has psychophysical properties that are observed during long-range apparent motion in vision, and during arpeggio playing in music. For example, if the ISI, or interstimulus interval, between the first and second tone decreases, then the traveling wave speeds up to smoothly interpolate the two tones. If the frequency difference between the two tones increases, but the ISI stays fixed, then the traveling wave again speeds up to smoothly interpolate the two tones. Since the second tone turns on only after the first tone turns off, these scaling properties raise interesting conceptual and philosophical questions that are settled by how a G-wave works.

The apparent movement from one tone to another allows us to enjoy music. In both vision and audition, it also has an important survival function: In vision, it enables our brains to continuously track a moving object, such as a prey or predator, as it runs with variable speed in a forest, while intermittently disappearing behind occluding cover. In audition, it enables our brains to continuously track a temporally discrete sequence of acoustically similar sounds, as during the performance of a piano sonata or a string quartet.

Our conscious recognition of a pitch percept is not fully modeled by the SPINET model. It is more fully modeled by the ARTSTREAM model (Figure 2, right panel) of how our brains can track multiple streams of sound through time, such as voices or instruments during music (Grossberg, 1999, 2021; Grossberg et al., 2004). The ARTSTREAM model incorporates the SPINET model as the front end of a larger neural architecture with enhanced capabilities, including conscious recognition of changing pitch sounds in an auditory stream. In ARTSTREAM, the event that supports conscious recognition of a pitch percept is modeled by a *spectral-pitch resonance* that creates an emergent *bound state* between a pitch category and the harmonic spectrum of sounds that it categorizes. Such a resonance emerges when the bottom-up adaptive filter that activates a learned pitch category within the Pitch Stream Layer triggers read-out of a top-down learned expectation back to the pitch’s frequency spectrum across the Spectral Stream Layer. When these bottom-up and top-down signals continue to cycle, they give rise to a resonant state between the pitch category and its harmonics. Although the pitch category is just a symbolic representation of the sound, the resonant bound state that it enables, supports a conscious percept of the sound spectrum.

Such a resonant state can drive learning in the bottom-up adaptive filter, leading to selective activation of its pitch category, and in the top-down expectation, leading to selective activation of the harmonics that support the pitch category’s activation.

The ARTSTREAM model also explains how a top-down expectation focuses attention upon the pitch’s harmonics while synchronizing and gain-amplifying their activation. It does this because each top-down expectation obeys the ART Matching Rule. The ART Matching Rule is embodied within a circuit that has been mathematically proved necessary to stabilize the learning and memory of any recognition category, including a pitch category (e.g., Carpenter and Grossberg, 1987, 1991).

A spectral-pitch resonance can flow through time between successively activated pitch categories as part of an auditory stream, as in the groupings described in Section 1.2 while discussing the Bach Partita.

An auditory stream is thus a percept that is caused when a G-wave is formed due to Gaussian receptive fields interacting across a topographically organized map of pitch categories in response to inputs to displaced positions across the map through time. One difference between apparent motion in vision and apparent motion in music is illustrated by the interactions between harmonics that occur during arpeggio playing. Due to these harmonic interactions, arpeggio playing of, say, the notes C E G C with increasing frequency, illustrates tonal music in which C E and G together form a “tonic triad” and end in the “tonic,” or key note, C.

### 1.4. Grouping by Working Memories and Learned Plans

In addition to grouping by pitch categories and auditory streams, our brains can temporarily store the sequences of words and notes that make up the lyrics and melodies of music, even before we can learn to perform them from memory.

The type of brain circuit that can temporarily store a sequence of events is called a *working memory*. Because a working memory can perform a stored sequence in the order that it occurs, it embodies a third kind of grouping. To date, it has been shown that a *single* canonical circuit design, suitably specialized, can store auditory, linguistic, spatial, and motor sequences in working memory, including sequences with repeated items, as in the sequence ABACBD. This type of Item-Order-Rank, or IOR, working memory will be described in more detail in Section 3. Lyrics and melodies, possibly with repeated words and notes, can both be stored in suitably specialized IOR working memories.

Sequences that are temporarily stored in working memory can be learned using list categories, which I also call *list chunks*. List chunks are a fourth kind of musical grouping. Just as harmonics can resonate with pitch categories in a spectral-pitch resonance, items that are stored in working memory can resonate with their list chunks in an *item-list resonance*. An item-list resonance supports learning of the list chunk that selectively categorizes the resonating list.

Although a spectral-pitch resonance supports conscious recognition of a pitch, it does not support conscious hearing of it. Conscious perception and recognition are each supported by different resonances. A *surface-shroud resonance* supports conscious seeing of a visual object or scene, and a *stream-shroud resonance* supports conscious hearing of an auditory object or stream. During vision, when a feature-category resonance and its corresponding surface-shroud resonance are simultaneously active, we can both consciously recognize and see the corresponding object. During audition, when a spectral-pitch resonance and the corresponding stream-shroud resonance are simultaneously active, we can both consciously recognize and hear the corresponding pitch.

These various resonances are part of a classification of the resonances that support conscious seeing, hearing, feeling, and knowing (or recognition) that are explained in Grossberg (2017b, 2021).

### 1.5. Issues Illustrated by Other Songs: Factorization of Order and Rhythm

These and related issues and processes are illustrated by the following two songs: The Alphabet Song^1^, and the song Smoke Gets In Your Eyes, with music by Jerome Kern and lyrics by Otto Harbach^2^.

English-speaking children typically learn the Alphabet Song: A B C D E F G (pause) H I J K (L M) (N O) P (pause),…. This notation connotes that each of the letters A, B,…, F and H, I, J, K is typically performed on a single beat, G and P are followed by a pause—that is, a silent beat with no letter performed—and the pairs of letters L, M and N, O are performed within a single beat. Moreover, the speed of performance can be volitionally increased or decreased without disrupting the relative timing of the letters. The letters can also be performed under volitional control with a different melody and/or rhythm. The Alphabet Song hereby raises questions about how a sequence of items is stored, learned, and performed in a given order, and with a prescribed melody and beat.

The beat is the steady pulse that you feel throughout a piece of music, like a clock’s tick, whether or not a musical note is played on any particular pulse. The rhythm is the actual pattern in time of the musical notes, which in a song also describes the times when the song’s words are sung.

The words of the Alphabet Song can be performed with different rhythms due to our brain’s ability to *factorize order and rhythm information*. The phrase factorization of order and rhythm denotes the fact that a sequence which is stored invariantly in an IOR working memory can be flexibly performed at a variety of rhythms under volitional control (Grossberg, 1986, 2003). Because of the factorization of order and rhythm, rate-invariant and speaker-invariant working memory representations (the “order”) can be flexibly performed in a rate-dependent and speaker-dependent way (the “rhythm”) that is under volitional control. In the special case of music, one must then explain how the lyrics that are stored in invariant working memories may be performed with different learned rhythms.

Such an invariant working memory representation both greatly reduces the amount of memory storage that is needed for storage, and makes it possible to learn the stored sequence’s meaning, which is coded by list chunks and their many learned associations throughout our brains. In contrast, were every language utterance stored in a rate-dependent and speaker-dependent way, then learning the meaning of one such representation would not generalize to any other representation. Indeed, learning from one teacher whose words are uttered with a given rate using a given frequency range (e.g., female) could not be understood when another teacher said the same words at a different rate or using a different frequency range (e.g., male). Language learning, among other skills, would then become impossible.

To avoid this catastrophe, order information is temporarily stored using a canonical IOR working memory circuit design whose specializations are capable of temporarily storing auditory, linguistic, spatial, or motoric sequences, including sequences with repeated sequences of letters such as ABACBD, repeated words in lyrics, or repeated pitches in songs. IOR working memories are used ubiquitously in our brains because they can quickly store, stably learn, and flexibly perform sufficiently short sequences of arbitrary kinds of information, including sequences that include repeated items (Grossberg, 1978a, 2017b, 2021; Grossberg and Pearson, 2008; Silver et al., 2011).

An example from speech illustrates the main idea that the process of factorization of order and rhythm embodies. You can ask: “How ARE you today?” or, just as easily, “HOW are YOU today?”, where the capital letters indicate a different rhythmic emphasis and duration. In past modeling analyses, this factorization property has been used to explain how speech can be performed with different rhythms (e.g., Grossberg et al., 1997a; Boardman et al., 1999). These studies immediately apply to performing the speech that constitutes lyrics with different rhythms. The current article extends this analysis to propose how lyrics and pitches can simultaneously be performed with different rhythms, including a regular beat that is generated in the basal ganglia; how learning and performance of more general, but still regular, rhythms is regulated by prefrontal cortical modulation of the beat; and how arbitrary performance rhythms are learned by adaptive timing circuits in the cerebellum as they interact with prefrontal cortex and basal ganglia. Moreover, the same type of circuit that controls beats also controls such basic motor skills as walking, running, and finger tapping.

### 1.6. Regular Rhythms, Counting, and Storing Repeated Words and Notes

The song *Smoke Gets in Your Eyes* begins with the phrase: “They asked me how I knew my true love was true.” The melody of this phrase poses two challenges to understanding how each brain learns and controls the performance of music. The first challenge is due to the fact that different words in the lyrics are performed with different timing. For example, the words “they,” “knew,” and the second occurrence of “true” are all held for four beats, while the remaining words are performed within one beat. How do we store, learn, and perform repeated words in a phrase with different timing? When learning to play this piece on an instrument like the piano, one strategy that piano teachers use is to ask their students to count the number of beats before the next note is played. That leads to the basic question: How do we count? A review of how humans count will be given in Sections 3.14 and 3.15.

A second challenging feature of the lyrics for *Smoke Gets in Your Eyes* is that the word “true” is repeated in two different places, each performed with different timing. These lyrics hereby illustrate the general cognitive problem of storing, learning, and performing sequences of items or events with repeated elements, such as ABACBD, and to do so with their own timing. Performing the same word more than once, with different timing at each occurrence, in a single song is a particularly good example of why I call our ability to do this *factorization* of order and rhythm.

### 1.7. Lyrics and Pitches Working Memories

**FIGURE 3:**
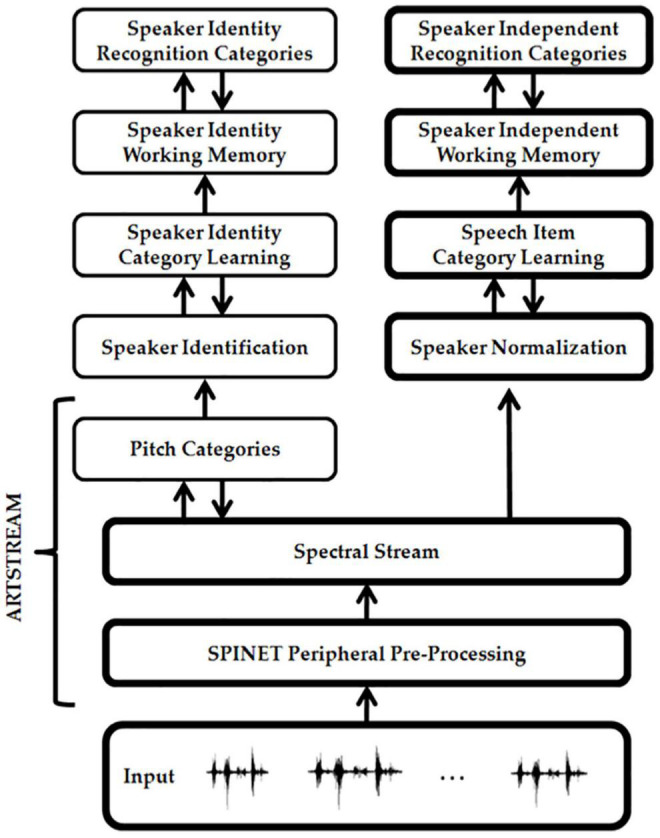
The ARTSPEECH architecture. ARTSPEECH consists of two parallel cortical processing streams, one devoted to speaker identification and the other to speaker meaning. Speaker identification can be learned from a speaker-dependent and rate-dependent representation of speech prosody. Speaker meaning can be learned from a speaker-normalized and rate-normalized representation of speech Item-Order-Rank information. Both streams process their distinct representations in working memories and reciprocally interact with chunking networks of similar design. [Reprinted with permission from Ames and Grossberg (2008)].

Multiple working memories exist in our brains to temporarily store sequences of different kinds of information. Figure 3 summarizes the ARTSPEECH model of two working memories that are needed for speech perception and recognition (Ames and Grossberg, 2008). This architecture illustrates the fact that working memories occur after a series of preprocessing stages. The rightmost stream includes a Speaker Normalization stage to convert speech to a *speaker-independent* form before speech item categories are learned and stored in working memory. A truly invariant working memory that could be used as a basis for learning language meaning would need to also preprocess incoming signals to make them *rate-invariant*. How rate-invariant speech representations are formed was modeled in Boardman et al. (1999) and Grossberg et al. (1997a). These mechanisms can readily be incorporated into the rightmost stream of Figure 3. The leftmost stream uses pitch categories to identify speakers.

**FIGURE 4:**
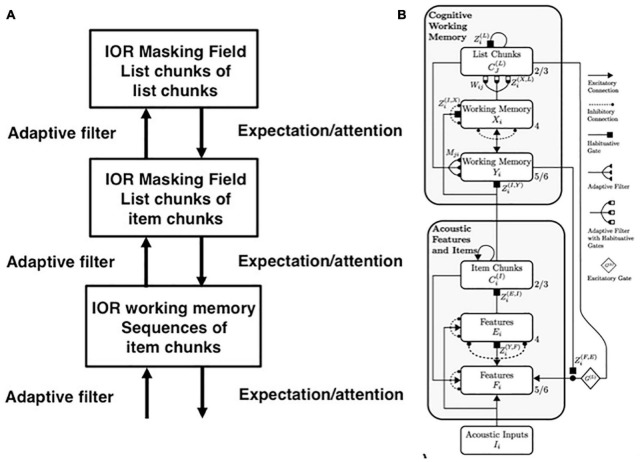
**(A)** Hierarchy of speech processing levels. Interactions among three speech processing levels are capable of working memory storage, learning, stable memory, and performance of word sequences with repeated words. Each level consists of an Item-Order-Rank working memory. The second and third levels are, in addition, multiple-scale Masking Fields that can store sequences of variable length. All the levels are connected by Adaptive Resonance Theory bottom-up adaptive filters and top-down learned expectations and their attentional focusing and memory stabilization capabilities. The first level stores sequences of item chunks. Its inputs to the second level enable that level to store list chunks of item chunks. The inputs of the second level to the third level enable it to store list chunks of list chunks, in particular sequences of words that may include repeated words. [Reprinted with permission from Grossberg and Kazerounian (2016)]. **(B)** Macrocircuit of the cARTWORD laminar cortical model for conscious speech perception shows a hierarchy of levels responsible for the processes involved in speech and language perception. Each level is organized into laminar cortical circuits. Deep layers (6 and 4) process and store inputs, whereas superficial layers (2/3) group distributed patterns across the deeper layers into categories, or chunks. The lowest level processes acoustic features (cell activities *F*_*i*_ and *E*_*i*_) and item chunks (cell activities $C_{i}^{\left(I\right)}$), whereas the higher level is responsible for storing of sequences of acoustic items in working memory (activities *Y*_*i*_ and *X*_i_), and representing stored sequences of these items as list chunks (activities $C_{j}^{\left(L\right)}$) in a Masking Field. [Reprinted with permission from Grossberg and Kazerounian (2011)].

Keeping in mind that multiple preprocessing stages are needed before any working memory in the brain is activated, let us return to a discussion of the working memories that are used to represent music. The IOR working memory that stores the sequences of words in a song is called a *lyrics working memory*. *List chunks* are learned from sequences of words that are stored in the lyrics working memory. List chunks are recognition categories that are selectively activated by specific sequences of words that are stored in working memory due to learned changes in the adaptive filter from the working memory level to the list chunk level (Figure 4A). The corresponding list chunks during speech perception are denoted by Speaker Independent Recognition Categories in Figure 3. Each list chunk can, in turn, learn to activate the sequence of lyrics words that it codes via learned changed in the top-down pathways from the list chunk level to the working memory level. These pathways are called top-down learned expectations (Figure 4A). As each lyrics list chunk is activated in its turn, it can use its top-down learned expectation to read-out its lyrics into working memory, from which they can be performed under volitional control.

A different working memory temporarily stores the sequence of pitches that constitute the melody of the song. This is the *pitches working memory*. The lyrics working memory and the pitches working memory are activated in parallel when listening to someone singing a song with those lyrics and melody. Just as the righthand stream of Figure 3 can represent lyrics, the lefthand stream of Figure 3 can represent pitches. As in the case of the lyrics working memory, a bottom-up adaptive filter from the pitches working memory can learn to activate pitches list chunks (cf. Speaker Identity Recognition Categories in Figure 3) and top-down pathways from a pitches list chunk to the pitches working memory can learn a top-down expectation whereby to read-out the sequence of pitches that it codes across the pitches working memory.

How these various processes work will be explained in Section 4. Additional learned associations enable the name of the piece of music, whether read via vision or heard via audition, to activate temporally ordered series of lyrics and pitches list chunks under volitional control. Volition is also needed to control finer aspects of performance. For example, the words that take up four beats in *Smoke Gets in Your Eyes* can be sung quickly−within a single beat that is followed by three beats filled with silence−or can be sustained throughout all four beats. This fact illustrates the distinction between the circuitry that controls the timed performance of the song as a whole, and the circuitry that modulates each word’s performance during the allotted timing using volitionally regulated breath control and emphasis.

## 2. Is Music Special?

### 2.1. An Analysis Based on Shared Brain Designs

A great deal has been written about how music may have emerged during evolution, how it compares with language, and how it has contributed to the development of social cognition, among other topics (e.g., Howell et al., 1991; Levitin, 2006; Thompson, 2009; Deutsch, 2013; Dowling and Tighe, 2014; Schulkin and Raglan, 2014; Tan et al., 2018). Essentially all of these observations have described psychological or neurobiological data about what happens during musical experiences.

The current article supplements this kind of descriptive knowledge with mechanistic neural explanations of how we learn and perform music. It hereby provides new insights about issues such as:

• Is music special to humans? If so, how?
• What similarities and differences exist between the neural mechanisms that control music perception and production vs. those that control language perception and production?
• How do musical rhythm and beat compare with other rhythmic activities?
• How can we perceive and perform music in different musical keys?

The discussion in Section 1 already touches on some of these issues. The remainder of the article will propose answers by describing brain design principles, mechanisms, and architectures that are needed to learn and consciously perform lyrics and melodies with variable rhythms and beats. It can thereby demonstrate how variations of the same brain design principles and mechanisms that control musical experiences are also used to accomplish other perceptual, cognitive, and motor competences than music. Although music has a unique place in our personal and cultural experiences−and has special properties due to its underlying harmonic structure−it also builds upon variations and specializations of other mental capabilities. Indeed, this article applies and specializes biological neural models that have been developed over the past 40 years to explain perceptual, cognitive, and motor processes other than music. Grossberg (2021) explains these concepts and models in greater detail and with many more scholarly references to other models and relevant data than I can present here.

## 3. Storing and Learning Event Sequences With Repeats: Working Memory and List Chunking

This section will review neural models of how working memories are designed and work in the brain. For reasons that will be explained below, the same kind of model circuit has successfully explained data about linguistic, motor, and spatial working memories. The lyrics of a song are an example of a linguistic working memory. The present article adds a pitches working memory to this list since pitches, no less than lyrics, obey the same laws that govern all of these types of working memory. This shared design of the working memories that encode lyrics and melodies enables our brains to coordinate the performance of a song’s words with its pitches using a prescribed rhythm.

### 3.1. Linking Working Memory Items and List Chunks

**FIGURE 5:**
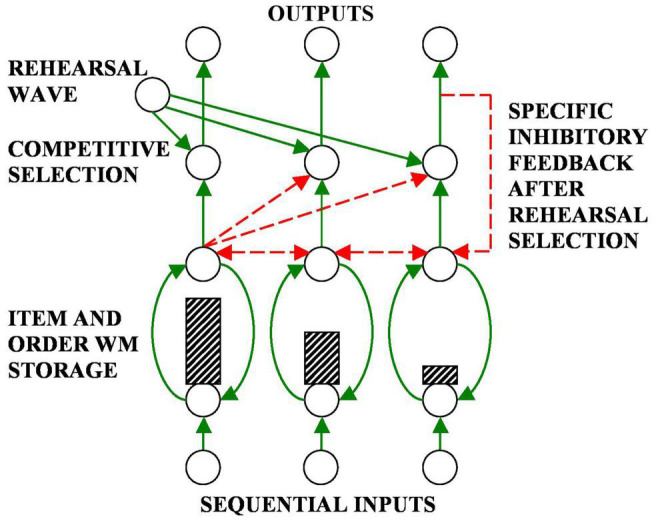
An Item-and-Order working memory is defined by a recurrent on-center off-surround network whose cells obey the membrane equations of neurophysiology, also called shunting laws (Hodgkin and Huxley, 1952; Hodgkin, 1964; Grossberg, 1973, 1980a; Carpenter, 1977a,b, 1979). Excitatory connections are in green. Inhibitory connections are in red. A primacy gradient of activity is stored in working memory in this figure (dashed rectangles denote relative cell activities). Two simultaneously converging inputs are needed to fire a competitive selection cell. One input is a specific input from the corresponding working memory cell. The other input is a nonspecific input called a rehearsal wave. The working memory cell with the largest activity can fire the corresponding competitive selection cell when a rehearsal wave is on. Its output signal also activates a specific inhibitory feedback signal that shuts the competitive selection cell off, and thus allows the next most active working memory cell to be rehearsed next. Competitive selection cells are called polyvalent cells in the subsequent exposition. [Adapted from Grossberg (2013a)].

The Item-and-Order neural model of working memory (WM) proposes that an incoming sequence of inputs that is received through time by our brains is stored as an evolving *spatial pattern* of item activities (Figure 5; Grossberg, 1978a,b). This name Item-and-Order model summarizes that its individual nodes, or cell populations, represent list *items*, while the temporal *order* of the items is stored by the spatial pattern of activity across the nodes. Item activities are sustained through time by recurrent excitatory neural signals from the item representations to themselves, balanced by recurrent inhibitory signals across multiple items; see Section 3.12. These stored spatial patterns of item representations are, in turn, unitized through learning into list chunk representations at the next processing level (Figure 4).

An item, or more precisely *item chunk*, selectively responds to prescribed patterns of activity across the distributed feature detectors within a prescribed time interval (e.g., a phoneme, musical note, or musical chord). A *list chunk* selectively responds to prescribed sequences of item chunks that are stored in working memory (e.g., a word or familiar grouping in the lyrics of a song). Thus, the item chunks of an Item-and-Order WM mediate between distributed feature patterns and list chunks. Properties of these functional units, interacting via bottom-up and top-down interactions, have been supported by their successful explanations and predictions of psychophysical data about speech perception, including immediate serial recall; immediate and delayed free recall; continuous-distracter free recall; long-term recency, word frequency, and word superiority effects; list length and list strength effects; presentation variability; phonemic similarity; and non-word lexicality (Grossberg, 1978a,b, 1984, 1986, 2003; Grossberg and Pearson, 2008). These functional units and their interactions will herein be used to explain how musical information is temporarily stored.

In particular, as will be explained below, just the three interacting processing levels shown in Figure 4A can store, learn, and perform lyrics that include repeats, such as “our true love was true.” This kind of example illustrates that our brains do not need, nor do they possess, many processing levels to store, learn, and perform sequential behaviors; cf. Figure 1 in Grossberg (2018).

### 3.2. Correct Temporal Order Is Stored in Working Memory by a Primacy Gradient

How does a spatial pattern that is stored in an Item-and-Order WM get performed in its correct temporal order? Performing musical notes in their correct order is, of course, essential in all musical performance. Correctly ordered performance occurs if the items in working memory are stored by a *primacy gradient* (Figure 5). For example, when a sequence “A-B-C” of items is stored by a primacy gradient, cells that store item ‘A’ have the highest activity, cells storing ‘B’ have the second highest activity, and cells storing ‘C’ have the least activity. Then the list ABC can be performed in the correct order because the item chunk with the highest activity is performed first, the item chunk with the second highest activity is performed second, and so on, until all items in the sequence are performed.

### 3.3. Rehearsal Waves and Inhibition-of-Return

**FIGURE 6:**
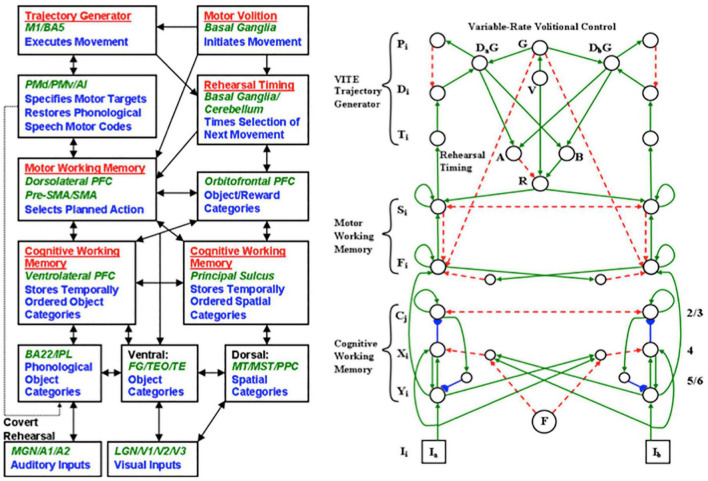
(left panel) The LIST PARSE laminar cortical model of working memory and list chunking includes circuits to model the brain regions that are marked in red. (right panel) The model’s Cognitive Working Memory circuit is proposed to occur in ventrolateral prefrontal cortex. The Motor Working Memory, VITE Trajectory Generator, and Variable-Rate Volitional Control circuits model how other brain regions, such as dorsolateral prefrontal cortex, motor cortex, cerebellum, and basal ganglia, interact with the Cognitive Working Memory to control working memory storage and volitional control of variable-rate performance of item sequences. [Adapted with permission from Grossberg and Pearson (2008)].

A primacy gradient that is stored in working memory does not have to be immediately performed. Performance occurs in response to a volitional signal that is called a *rehearsal wave*. The basal ganglia where the volitional signal originates has no knowledge about what is stored in working memory in prefrontal cortex, or PFC (Figure 6). A rehearsal wave is therefore delivered uniformly, or nonspecifically, with equal activity from the basal ganglia to the entire PFC WM (Figure 5). The rehearsal wave enables read-out of stored activities by opening a rehearsal gate. The item chunk with the highest activity is read out fastest because it exceeds its output threshold fastest. Its output signal also self-inhibits its WM representation via a specific inhibitory feedback pathway (Figure 5), leading to *inhibition-of-return* that prevents perseverative performance of the most active item (Grossberg, 1978a,b; Posner and Cohen, 1984; Posner et al., 1985; Klein, 2000). Each item representation self-inhibits as it is rehearsed until no active items are left in WM.

### 3.4. Item-and-Order, Competitive Queuing, and Primacy Models

Since the Item-and-Order WM model was introduced, many modelers have applied it (e.g., Houghton, 1990; Boardman and Bullock, 1991; Bradski et al., 1994; Page and Norris, 1998; Bullock and Rhodes, 2003; Grossberg and Pearson, 2008; Bohland et al., 2010). For example, Page and Norris (1998) have used a Primacy Model to explain cognitive data about word and list length, phonological similarity, and forward and backward recall effects. Houghton (1990) called the model Competitive Queuing when he also used it to explain cognitive data.

### 3.5. Item-and-Order Model Explains Psychological and Neurophysiological Data

Subsequent psychophysical and neurophysiological experiments confirm that, as predicted, item order is encoded by relative activity levels and is reset by self-inhibition. For example, Farrell and Lewandowsky (2004) studied the latency of human responses that follow serial performance errors. They found that (p. 115):

“Several competing theories of short-term memory can explain serial recall performance at a quantitative level. However, most theories to date have not been applied to the accompanying pattern of response latencies…Data from three experiments…rule out three of the four representational mechanisms. The data support the notion that serial order is represented by *a primacy gradient that is accompanied by suppression of recalled items* [italics mine].”

**FIGURE 7:**
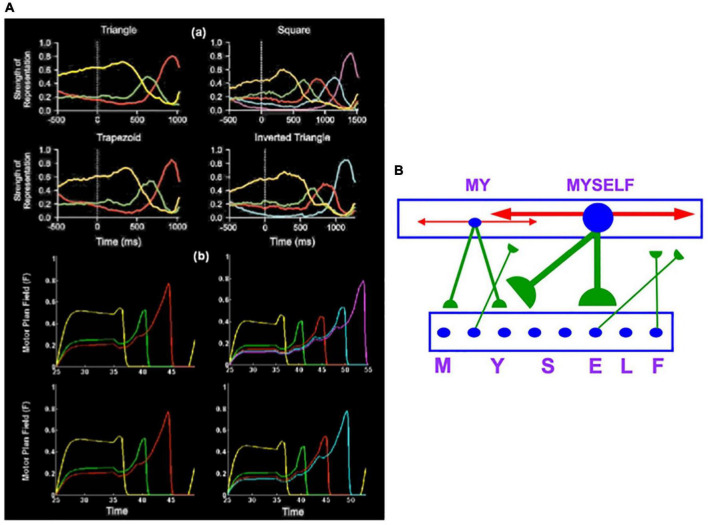
**(Aa)** Neurophysiological data that conform to Item-and-Order working memory properties were recorded in a series of sequential copying experiments in monkeys [Adapted with permission from Averbeck et al. (2002)]. Each of the four figures shows a primacy gradient in working memory whose most active cell is performed first as its activity self-inhibits, followed by the next most active cell, and so on. When only the last item remains, it has the highest activity because it was freed from inhibition by earlier items. **(Ab)** The LIST PARSE laminar cortical model of working memory and list chunking simulates these data. [Reprinted with permission from Grossberg and Pearson (2008)]. **(B)** Circuit illustrating how Item-and-Order stored working memory item chunks (M, Y, S, E, L, F) activate list chunks (such as MY and MYSELF) in a Masking Field network. Masking Field cells respond selectively to lists of item chunks of variable length. How this happens is summarized in the text. [Reprinted with permission from Grossberg (2020b)].

Electrophysiological experiments of Averbeck et al. (2002) studied macaque monkeys performing arm movement sequences that copy geometrical shapes. The data curves at time zero in the four graphs in Figure 7Aa exhibit the primacy gradients of four lists that were stored in dorsolateral prefrontal cortex. These curves also show that the most active cells are read-out earliest and self-inhibit to permit read-out of the entire list in the stored order. The data were simulated (Figure 7Ab) by an Item-and-Order working memory in the LIST PARSE laminar cortical model (Figure 6; Grossberg and Pearson, 2008). These properties also occur in the Item-Order-Rank, or IOR, generalization of Item-and-Order working memories that can temporarily store sequences of pitches that may include repeated notes, as in the first four phrases of Bach’s Partita #1; see Figure 1 and Section 3.14. Figure 7A shows that LIST PARSE successfully models a dorsolateral prefrontal *motor* working memory that can quantitatively simulate neurophysiological data about sequential recall of stored motor sequences. LIST PARSE was also shown in Grossberg and Pearson (2008) to model a prefrontal *linguistic* working memory in the ventrolateral prefrontal cortex that quantitatively simulates psychophysical data about immediate serial recall, and immediate, delayed, and continuous distractor free recall, among other data properties.

### 3.6. Bowed Gradients, Grouping, and Chunking

Not all sequences of items that are stored in a working memory can be recalled in their correct temporal order. Only primacy gradients have this property. How and when primacy gradients occur thus clarifies how music is typically performed in the correct temporal order.

Free recall tasks illustrate how a sequence of items can be performed in an incorrect order. During free recall, a list is recalled in whatever order comes to mind after hearing it just once (e.g., Murdock, 1962). If the stored list is too long, a *bowed* serial position curve is often observed. Here, items at the beginning and the end of the list are recalled earliest, and with the highest recall probability.

Grossberg (1978a,b) noted that these free recall properties have a natural explanation if the pattern of cell activities that stores the list items in working memory is also bowed, with the first and last item chunks having the largest activities, while those in the middle having less activity. Then, the item chunk with the largest activity is read out first, whether at the list’s beginning or end, and self-inhibits its item representation to prevent preservation (Figure 5). Then the next largest item chunk is read out, and so on in the order of stored relative activity.

As in free recall data, items at the beginning and end of the list are recalled with greater probability because larger activities can better survive perturbations due to internal cellular noise and attentional fluctuations. *Transpositions* of recall order by items close together in the list are explained by the fact that they have similar stored activities, so their relative size, and thus temporal order, can more easily be reversed by internal noise or attentional fluctuations.

These facts about when primacy or bowed gradients are stored in working memory constrain strategies for storing all sorts of sequences in working memory, including lyrics and pitches, so that they can be learned and performed in the correct order. To understand this issue better, answers will be provided to the following questions:

• What are the longest lists that our brains can store in working memory in the correct temporal order?
• Why can only relatively short lists be stored with the correct temporal order?

In an Item-and-Order working memory, these questions become:

• What is the longest primacy gradient that the working memory can store?
• Why is it so short?

### 3.7. Memory Spans Constrain Musical Groupings: The Magical Numbers Four and Seven

What is the longest primacy gradient that can be stored? The answer to this question constrains all strategies for learning to correctly perform skilled sequences, whether during speech production, dance movements, spatial navigation, or musical performance. The upper bound during free recall has been called the Magical Number Seven, or *immediate memory span*, of 7 ± 2 items (Miller, 1956).

The explanation in Grossberg (1978a) of the immediate memory span distinguished it from the then new concept of *transient memory span*. The transient memory span was predicted to be the longest list for which a primacy gradient may be stored in short-term memory solely as the result of bottom-up inputs, and without the benefit of learned expectations being read-out top-down from active list chunks. The immediate memory span, in contrast, was predicted to arise from the combined effect of bottom-up inputs and top-down read-out from learned expectations.

My article Grossberg (1978a) proved that read-out of top-down expectations can only increase the maximal primacy gradient that can be stored, thereby predicting that the immediate memory span exceeds the transient memory span. Given the known estimated immediate memory span of approximately seven items, the transient memory span was estimated to be approximately four items. There should thus also be a Magical Number Four when top-down effects are removed. This prediction was experimentally supported by data of Cowan (2001) who demonstrated a working memory capacity of 4 ± 1 items when influences of long-term memory and grouping effects were minimized in his experimental design.

The Magical Numbers Four and Seven for storage of items in working memory shed mechanistic light on the maximum length of musical groupings that can readily be performed in the correct order. In particular, these constraints clarify how the small number of notes in each of the groupings that occur in Bach’s Partita Number 1 (Figure 1) facilitate how this exquisite piece of music is stored, learned, and performed in the correct order.

### 3.8. LTM Invariance Principle: Working Memory Supports Stable List Chunk Learning

Why is the transient memory span so short? My answer to this question is, basically, that it does not pay to store item sequences in working memory if they cannot be learned. In other words, temporary storage of sequences in working memory is useful only if it can support stable learning of list chunks, and read-out during performance by those list chunks of the sequences in working memory that they code. In the case of music, list chunks need to be learned from both lyrics working memories and pitches working memories. It will also be shown below how list chunks that encode rhythms are learned.

Upon realizing the paramount importance of learning, in Grossberg (1978a,b). I *derived* Item-and-Order working memory circuits from hypotheses that ensure their ability to support learning and stable memory of list chunks. When this insight is applied to music, it clarifies that musical groupings are short both to store them in working memory for possible immediate performance in the correct temporal order, as well as to chunk them via learning for future performance in that order.

Item-and-Order working memories embody two simple postulates that enable their list chunks to be learned and stably remembered: the LTM Invariance Principle and the Normalization Rule. These postulates were used to derive mathematical equations for Item-and-Order working memories, and to mathematically prove how they generate primacy and bowed gradients.

The LTM Invariance Principle prevents storage of longer lists of events in working memory (such as MYSELF) from causing catastrophic forgetting of previously learned list chunks of shorter lists (such as MY, SELF, and ELF). In particular, if bottom-up inputs activate a familiar list chunk, such as the word MY, then storing in working memory the remaining portion SELF of the novel word MYSELF during the next time interval will not cause forgetting of the learned weights that activate the list chunk of MY. When applied to music, this Principle enables larger groupings of notes to be learned without forgetting previously learned smaller groupings.

Incremental refinements of these models have been made over the years (e.g., Bradski et al., 1992, 1994), eventually leading to models of how the layered circuits in the prefrontal cortex compute IOR working memories, among other properties necessary to achieve higher-order properties of biological intelligence (Grossberg and Pearson, 2008; Silver et al., 2011; Grossberg, 2013a, 2018, 2021).

### 3.9. Stable List Chunking Exploits Classical Laws for Adaptive Filtering and Competition

Stable list chunks can be learned because, as new inputs are stored in working memory, the *relative activities*, or ratios, of previously stored working memory activities are preserved, even if the newly arriving inputs may change their total activities. As a result, the relative activities of previously learned *adaptive weights*, or LTM traces, are also preserved. This happens because the bottom-up signals in the axons from the working memory to the list chunks are *multiplied* by the LTM traces before the net signals activate list chunks.

For example, in the *conscious ARTWORD*, or cARTWORD, model of conscious speech perception (Figure 4B; Grossberg and Kazerounian, 2011), the LTM traces are computed in synaptic knobs at the ends of bottom-up axons, in abutting postsynaptic membranes, or both. These synaptic knobs are represented by black-filled hemidisks in Figure 4B. The open squares abutting the hemidisks denote that these synapses can habituate in an activity-dependent way. A habituated synapse releases less chemical transmitter than an unhabituated one in response to an input signal of a fixed size. Habituation helps the network to reset itself in response to new inputs, so that old responses do not perseverate for too long.

The bottom-up adaptive signals from multiple axons are added up at each recipient list chunk. The total input to a list chunk thus multiplies a *pattern*, or vector, of activities times a *pattern*, or vector, of LTM traces. By preserving relative activities, the relative sizes of these total inputs to the category cells do not change through time; thus, nor do the corresponding LTM patterns that track these activities when learning occurs at their category cells. That is why SELF does not recode a previously learned category for MY when MYSELF is presented through time. The bottom-up LTM-gated pathways from the working memory to the list chunking level constitute the *adaptive filter* pathways depicted in Figure 4.

The words MY, SELF, and MYSELF can be replaced by the words in lyrics or the pitches in a melody. Larger groupings of musical elements can hereby be learned without forcing forgetting of previously learned subgroupings of them.

The Normalization Rule means that the total WM activity has a maximal value that is approximately independent of the number of stored items. Thus, storing more items in WM forces each item to be stored with less activity. As storage of more items in working memory converts a primacy gradient into a bowed gradient, normalization forces the stored item activities to become smaller. Normalization mechanizes the *limited capacity* of WM.

An adaptive filter can activate multiple cells that code list chunks. These cells compete to choose a winning cell, or small set of cells, that receive the largest inputs (Figure 7B). The winning cells code list chunks that have the most support from their bottom-up inputs in the current context. Winning cells drive learning whereby their abutting LTM traces track the bottom-up input patterns that they filter. This adaptive tuning process enables them to fire more selectively to the input patterns that activated them, leading to the name *competitive learning* for this kind of category learning network (Grossberg, 1976a,b, 1978a; Willshaw and von der Malsburg, 1976; Rumelhart and Zipser, 1985).

### 3.10. Masking Fields Can Learn and Perform Musical Groupings of Variable Length

The example of MY and MYSELF illustrates that list chunks can selectively represent lists of variable length in order to learn language, music, or motor skills like dancing, playing the piano, and navigating routes in space. The category cells that occur in Masking Field networks support learning and storage of *variable-length* list chunks (Figure 7B). To accomplish this, a Masking Field consists of a multiple scale, self-similar, recurrent shunting on-center off-surround network (Cohen and Grossberg, 1986, 1987).

Masking field cells develop with multiple sizes, or scales, due to activity-dependent growth during development. During development, item chunks are endogenously active during a critical period, leading to the growth of bottom-up connections from the item chunk level to the list chunk level. These connections grow accordingly to a probabilistic law whereby variable numbers of connections contact list chunks across the network. List chunks that receive bottom-up inputs from more item chunks can code longer lists. They also receive larger total inputs, on average, through time. During the network’s development, input activity triggers self-similar cell growth whereby both cell bodies and their connections grow proportionally. This growth continues until the total activity density is reduced to a threshold intensity. The net result is a Masking Field wherein longer lists are coded by larger cells with stronger recurrent inhibitory interneurons within the list chunk level (red connections in Figure 7B), and stronger top-down excitatory priming pathways to the item chunk level (green connections in Figure 7B).

Masking Field nodes are list chunks in the second and third processing levels in Figure 4A. When representing language or lyrics, the first level can represent letters, the second level words, and the third level sequences of words. As noted above, because the second and the third levels are also Item-Order-Rank working memories, the words coded at the second level can include repeated letters, as in the words “repeated” and “letters,” and the sequences coded at the third level can include repeated words, as in the phrase “our true love was true.” The same is true for pitches. Then the first level can code musical notes or chords, the second level can code short pitch sequences that may contain repeated chords, and the third level can code sequences of pitch phases in a melody.

### 3.11. A Universal Design for Linguistic, Motor, Spatial, and Musical Working Memories

If all linguistic, motor, spatial, and musical working memories obey the LTM Invariance Principle and the Normalization Rule, then they should all share a similar design. Both psychological and neurobiological data support this prediction. Models that explain and simulate linguistic, motor, and spatial working memory data include the laminar cortical LIST PARSE model [Figure 6 (right panel); Grossberg and Pearson, 2008] that uses a prefrontal *linguistic* working memory to explain and quantitatively simulate psychophysical data about immediate serial recall, and immediate, delayed, and continuous distractor free recall. Note the cortical layers 5/6, 4, and 2/3 in the Cognitive Working Memory in Figure 6 (right panel). LIST PARSE also describes a prefrontal *motor* working memory that quantitatively simulates neurophysiological data about sequential recall of stored motor sequences (Figure 6, left and right panels).

The lisTELOS model (Silver et al., 2011) incorporates LIST PARSE as a prefrontal *spatial* IOR working memory that quantitatively simulates psychological and neurophysiological data about the learning and planned performance of saccadic eye movement sequences. lisTELOS models how several parietal and prefrontal cortical areas interact together and with three basic ganglia gating circuits. These cortical areas include the Supplementary Motor Area, or SMA, and pre-SMA, whose damage degrades performance of stored sequences in working memory (Shima and Tanji, 2000; Kennerley et al., 2004; Zatorre et al., 2007; Nachev et al., 2008). Due to the homology between linguistic, motor, and spatial working memories, the results apply to any sequentially organized behaviors, including musical behaviors.

### 3.12. Recurrent Shunting On-Center Off-Surround Networks Embody Working Memories

The LTM Invariance Principle and Normalization Rule are realized by a type of circuit that occurs ubiquitously throughout our brains. It is a recurrent on-center off-surround network of cells that obey the membrane equations of neurophysiology, otherwise called shunting dynamics (Figure 5). How these networks process ratios (LTM Invariance Principle) and conserve total activity (Normalization Rule) was mathematically proved in Grossberg (1973); also see reviews in Grossberg (1978a, 1980b, 2013b, 2021).

In such a network, excitatory feedback due to recurrent on-center interactions (green arrows in Figure 5) helps to store an evolving spatial pattern of activities in response to a sequence of inputs, while recurrent off-surround shunting interactions balance the on-center to store input relative activities (horizontal red arrows in Figure 5), thereby generating the desired properties of contrast normalization and conservation of total activity.

A rehearsal wave from the basal ganglia (Figure 6) reads-out the highest stored activity first, and self-inhibitory feedback prevents its perseverative performance (Figure 5), while the network gradually renormalizes its activity through time.

The effects of recurrent inhibition can be seen in the data and simulation summarized in Figure 7A: After the next-to-last item is performed, the cell activity that stores the last item is no longer inhibited by other cells, so it becomes more active than previously active cells.

### 3.13. Recurrent Shunting Networks Also Control Beat and Gamma Oscillations

Remarkably, the oscillatory circuits that control musical beat (see Section 5) are also recurrent on-center off-surround networks with cells that obey shunting dynamics. What distinguishes them from working memories are the following kinds of differences:

First, the beat oscillator does not include self-inhibitory interneurons to prevent cyclic performance (Figure 5), although they may include self-inhibitory interneurons as part of a recurrent shunting off-surround.

Second, and more important, the beat oscillator is driven by a sufficiently large arousal, or GO, signal that converts it from a phasically responsive network to one that oscillates, much as the same corticospinal circuitry can regulate standing and walking. Sufficiently aroused working memories can also oscillate, albeit with faster alpha, beta, gamma, and theta oscillations, during perceptual and cognitive processing (Grossberg, 2017a,b, 2021). This kind of oscillator can thus support a range of oscillatory periods, depending upon parameter choices.

### 3.14. Item-Order-Rank Coding: Numerical Hypercolumns Store Lists With Item Repetitions

How do working memories store lists with repeated items? This competence is needed to store lyrics with repeated words, and melodies with repeated pitches. For this to occur, a working memory must be able to selectively store items with sensitivity to their list position, or *rank*.

Cognitive data demonstrating sensitivity to rank include spoonerisms, during which words or syllables in similar positions, but in different words, are interchanged; e.g., “hissed my mystery lesson” (Henson, 1998). Neurophysiological data from cells in prefrontal cortex also exhibit rank sensitivity (Barone and Joseph, 1989; Kermadi and Joseph, 1995; Funahashi et al., 1997; Averbeck et al., 2003: Ninokura et al., 2004; Inoue and Mikami, 2006).

**FIGURE 8:**
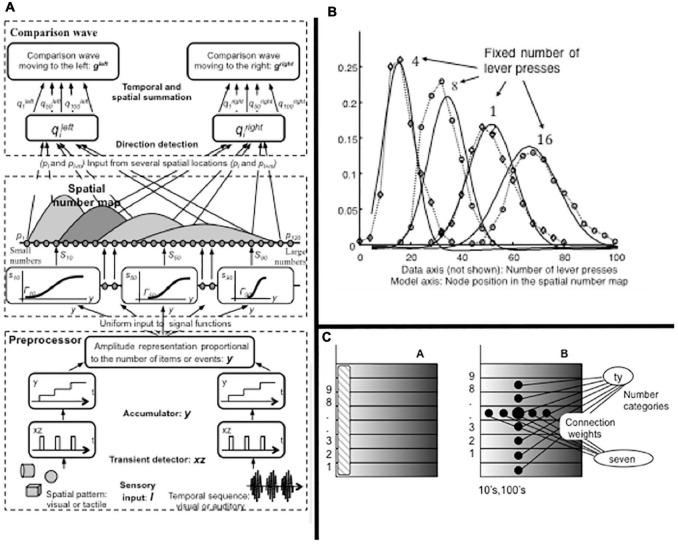
**(A)** The SpaN model simulates how spatial representations of numerical quantities are generated in the parietal cortex. See text for details. **(B)** Behavior numerosity data and SpaN model simulation of it. The responses to increasingly large numerical inputs activate different, but contiguous, positions in the analog number map, with larger numbers generating responses further to the right on the map. The larger variance of responses to larger numbers is called the Weber Law. All these properties obtain in both the data and the model simulation. **(C)** The Extended SPaN, or ESpaN, model extends SpaN to be able to learn and perform place-value numbers. Interactions from the What cortical stream to the Where cortical stream accomplish this extension. In particular, previously learned phonetic categories in the What stream become associated with corresponding locations of the SpaN spatial number map in the Where stream. **(CA)** The striped area shows the location of the primary number map. This is extended into parallel strips all of whose cells also respond to the inputs to the primary number map. **(CB)** An example of where the association for *seven-ty* is learned in this strip map. The size of the solid circles encodes weight magnitude; the strongest association for *seventy* is arises at the spatial location where both the associations for categories *seven* and *ty* are present. [Reprinted with permission from Grossberg and Repin (2003)].

How is rank information incorporated into an Item-Order-Rank working memory model that can store item repeats at arbitrary list positions; e.g., ABACBD? Grossberg and Pearson (2008) proposed that an Item-Order-Rank working memory in prefrontal cortex derives rank selectivity via a parietal-to-prefrontal projection from the analog number map that exists in parietal cortex. This prediction built upon a model of the parietal number map called the Spatial Number Network, or SpaN, model (Figure 8; Grossberg and Repin, 2003). SpaN simulates how the parietal number map may control the ability of animals and humans to estimate and compare small numerical quantities without requiring that they count these quantities with numbers (Rickard et al., 2000; Naccache and Dehaene, 2001).

Figure 8A summarizes how SpaN works. Each event in a sequence activates a transient detector that generates an input burst (see the series of rectangular inputs denoted by xz). These bursts are added to an accumulator (see the series of steps denoted by y). This stored total input is broadcast uniformly across the *spatial number map*. Cells at different positions in the number map have thresholds and sensitivities that increase from left to right across the map (see the sigmoid signal functions). As a result, small inputs selectively activate cells near the left of the map, whereas larger inputs activate positions to the right. Increasingly large inputs activate a series of unimodal response profiles centered at positionally displaced positions toward the right.

Figure 8B shows the close fit of the model’s response profiles to numerosity data collected during animal conditioning experiments. The response profiles of SpaN model parietal neurons are also matched by neurophysiological data of Nieder and Miller (2004a).

Nieder and Miller (2004b) also reported a prefrontal projection *in vivo* from the parietal number map. In the SpaN model, this parietal-prefrontal projection uses the ordered number map in the parietal cortex to embed numerical *hypercolumns* within the prefrontal working memory. This prediction has not yet been tested using neurophysiological methods.

**FIGURE 9:**
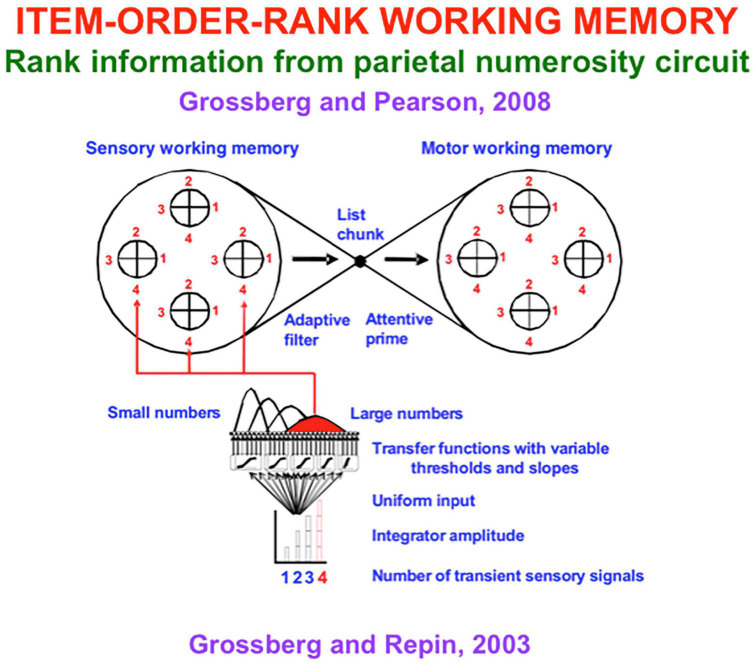
Circuit for encoding a conjunction of item, order, and position in sensory and motor working memory. Cells in the sensory and motor working memories need a second input that codes positional information in order to fire. The model proposes that number maps in parietal and frontal cortex provide this positional information. The circles with numbers represent cortical hypercolumns, each coding a different sensory or motor event, with positions (for illustration) 1, 2, 3, and 4. The sensory working memory supports learning of list chunks. The list chunks learn to attentively prime the Item-Order-Rank motor working memory during reactive performance of a sequence of actions. During planned performance, cells in the motor working memory fire their motor commands when they receive a list chunk priming signal and the correct positional, or rank, input from the corresponding number map. The lower part of the figure illustrates how transient inputs in response to each sensory event are integrated into a signal proportional to the total number of sensory inputs that have occurred in the sequence. This integrated signal generates a uniform input to all the cells in the parietal number map. The signal functions with variable thresholds and slopes in the number map cause distinct populations of cells to get activated as a larger number of transients is stored. The number map cells broadcast their positional information to the sensory working memory. A similar scheme occurs in the motor working memory. [Reprinted with permission from Grossberg and Pearson (2008)].

Figure 9 describes how the parietal number map projects to hypercolumns in the prefrontal cortex (see red activity profile and red pathways). Each item in the list is stored in a different position in its hypercolumn if it is repeated more than once. Each item’s hypercolumn representation is denoted by a circle in Figure 9, and each of its four hypercolumn positions is denoted by a pie-shaped region within this circle and is numbered from 1 to 4.

For example, consider how a numerical hypercolumn can store and perform in its correct order the short list ABAC: Item A is stored in positions 1 and 2 within its hypercolumn, item B is stored in position 1 within its hypercolumn, and item C is stored in position 1 within its hypercolumn. A primacy gradient of activity can store the temporal order of a short list, whether or not it has repeated items. In response to the list ABAC, for example, each of the successively activated map positions would have a progressively smaller activity stored in working memory. The recurrent on-center off-surround network that stores items in such an Item-Order-Rank working memory can still have self-excitatory feedback from each cell population to itself, and a broad off-surround that inhibits all other populations equally across the hypercolumns. When self-inhibitory feedback inhibits the last-performed item, the next item is performed, as usual, until the entire list ABAC is performed.

The numerical hypercolumns are represented by segmented circles in Figure 9 for convenience. They are more realistically represented by strips in a cortical map. Taken together, the totality of these strips across a cortical region provides our first example of a *strip map*.

### 3.15. Keeping Time by Counting: Place-Value Numbers

Sometimes we keep time in music by counting using numbers. Grossberg and Repin (2003) showed how number names may get associated with the spatial number map (Figure 8C). First, number names get learned in the What, or ventral, cortical stream as part of language development and learning. Then these number names get linked via associative learning with a corresponding spatial numerical map representation in the Where, or ventral, cortical stream. Grossberg and Repin (2003) also explained and simulated how this associative What-to-Where map can learn representations of larger numbers, called *place-value numbers*, such as “twenty,” “thirty,” or “one hundred.” To accomplish this extension, the primary spatial number map (shown as a vertical striped bar in Figure 8CA) is extended to a set of horizontal strips, each of which extends the representation of its number in the primary map. Then associative learning from the number names to the number map takes place (Figure 8CB), as illustrated by the number seventy. These horizontal strips constitute another example of a strip map, a general brain design that will be seen below to have multiple functions in music.

### 3.16. Strip Maps: A Cortical Design With Multiple Uses in Music

Both place-value numbers and working memories with repeated items are coded in the cerebral cortex using strip maps. Indeed, strip maps occur throughout our brains. The orientation columns within cortical area V1 are the most famous example of strip maps. Here each strip, or hypercolumn, includes map positions that respond selectively to objects with different orientations at the position that it codes, and the entire cortical map contains multiple strips that together are sensitive to all visible positions (Hubel and Wiesel, 1962, 1963).

In general, a strip map represents one feature throughout its extent (e.g., position), as well as another feature in an ordered array of positions throughout the strip (e.g., orientation). In addition to strip maps that represent orientation columns, place-value numbers, and cognitive working memories that can code repeated items, strip maps also occur in models of auditory streaming and speaker-normalized speech (Grossberg et al., 2004; Ames and Grossberg, 2008).

All of these strip maps are relevant to music. For example, auditory streams can separate and track the instruments that we hear during a string quartet. Place-value numbers can be used to identify a piece of music, such as the BWV (Bach Works Catalogue) number of a particular piece of music by Bach, or the page number of a particular composition in a book of music. The exposition in the next section will show how specialized strip maps can also represent musical lyrics, pitches, and rhythms.

## 4. Storing, Learning, and Performing Musical Lyrics, Pitches, and Rhythms

### 4.1. Storing and Performing a Phrase of Lyrics During Bidirectional Learning With Its List Chunk

This section will propose how a grouping of lyrics or melody can be stored in working memory and performed with a regular rhythm whose delays can be learned by counting the beats between notes. It is proposed how the counting process for one word or note delays the performance of the next word or note for the correct duration.

This explanation builds upon the fact that an Item-Order-Rank working memory (IOR WM) can use numerical hypercolumns to store words or notes that may occur in multiple positions within a short enough grouping; e.g., “our true love was true.” To be learned and performed in the correct order, a grouping of lyrics or notes must be short enough to be stored as a primacy gradient (Figures 1, 5, 7A).

**FIGURE 10:**
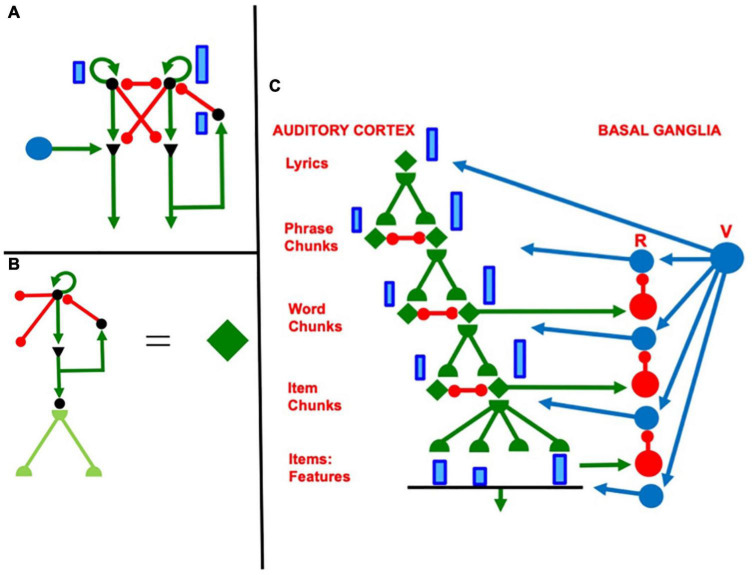
**(A)** When a rehearsal wave R (blue disk) turns on, the item that is stored in working memory can be rehearsed while it self-inhibits its working memory representation. Relative activity amplitudes are represented by the sizes of vertical blue rectangles. Triangular cells are polyvalent. **(B)** Filled diamond summarizes key stages in choosing items to be rehearsed and associating them with bottom-up adaptive filter and learned top-down expectations. **(C)** Recursive read-out, under volitional control, from the hierarchy of processing stages that represents the lyrics of a song. Green represents excitatory connections. Red represents inhibitory connections. Blue disks represent volitional gain control signals. See the text for details.

In Figure 5, the rehearsal wave acts at a processing stage that occurs after the items that are stored by a WM gradient compete to choose the largest activity. A rehearsal wave allows this winning activity to be read out for performance, even as it self-inhibits its WM representation using a specific inhibitory feedback pathway. Figure 10 begins to use the above foundation to explain how the lyrics of a song can be performed with a learned and possibly variable rhythm, as illustrated by the songs described above. The same mechanisms and circuits can be used in a parallel architecture to explain how the melody of a song can be performed along with the lyrics.

Figure 10A repeats the circuit in Figure 5 using a notation that will be convenient for representing a larger cognitive architecture in the auditory cortex whose rhythmic performance is regulated by volitional control from the basal ganglia and cerebellum. In Figure 10A, excitatory connections are depicted by green arrows and inhibitory connections are depicted by red connections that end in red disks. The two cells that are represented by black disks at the top of Figure 10A interact via a recurrent shunting on-center off-surround network and can thus store an activity pattern in WM. The blue vertical bars of unequal height illustrate the activity pattern that is currently stored in WM by this network. Everything that is written below generalizes to networks with an arbitrary finite number of cells.

The WM network in Figure 10A outputs via a non-recurrent, or feedforward, on-center off-surround network to a pair of cells that are denoted by black triangles. This feedforward competitive network chooses the larger activity that is stored in WM for further processing, while inhibiting the smaller activity. This competition results in allowing only one cell at the next processing stage to receive a positive input; namely, the cell beneath the one that has been storing the larger activity in WM.

The black triangles denote polyvalent cells that can fire only when they receive converging inputs from specific and nonspecific input sources. The specific input comes from the WM network. The nonspecific input comes from the blue cell (population). Activating this blue cell occurs when rehearsal is desired. Activating the blue cell releases a rehearsal wave that is represented by the horizontal green arrow. Only one of its excitatory output pathways is shown. In fact, the blue cell sends equal excitatory signals to all of the polyvalent cells whenever it fires. It is thus a source of *nonspecific arousal*.

The polyvalent cell that receives both a specific and a nonspecific input can then fire. It then can send a signal along its output pathway, which is denoted by a downward facing green arrow. This signal also activates a recurrent specific inhibitory interneuron, which shuts off the WM cell that activated it. When this WM is silenced, so too are its feedforward inhibitory signals that competitively silence outputs from the rest of the WM network. Because the rehearsal wave is brief, all additional outputs from the WM network are prevented.

The circuit on the left hand side of Figure 10B summarizes a single cell in the WM network and all of its output connections. This unit will be repeated multiple times in larger cognitive architectures. In order to facilitate drawing such architectures, it is denoted in various other figures as the filled green diamond on the right hand side of Figure 10B. The left hand side of Figure 10B also includes bidirectional adaptive pathways at the next processing stage. These pathways are drawn in light, rather than dark, green because the rest of the circuit with which these adaptive pathways interact is not shown. Both the bottom-up and top-down pathways are adaptive and are thus denoted by green hemidisks. This notation will also be used in larger architectures.

### 4.2. Learning Lyrics in a Hierarchical Cortical Architecture: Recursive Read-In

Previous articles have modeled how sequences of items that are stored in WM can be encoded by learned list chunks at the next processing stage (Bradski et al., 1994; Grossberg and Kazerounian, 2011, 2016; Kazerounian and Grossberg, 2014). A volitional gain control source initiates the storage process. This gain control process can act iteratively to learn a hierarchy of ever-more-complex sequential representations. Figure 4A summarizes a three-level processing hierarchy that can learn phrases and sentences with repeated items. The volitional gain control process that regulates such storage and learning was omitted from this figure, but can be studied in the articles cited above. The top level in Figure 4A corresponds to the Phrase Chunks level in Figure 10C.

### 4.3. Rehearsing Lyrics From a Hierarchical Cortical Architecture: Recursive Read-Out

Figure 10C incorporates the network components in Figures 10A,B into a hierarchical cortical architecture that can represent WM storage and fluent read-out of the lyrics of an entire song using multiple cortical processing areas. The top-most list chunk in this figure is a Lyrics Chunk. When its top-down learned expectation pathways are activated by a rehearsal wave R, it reads out Phrase Chunks that are stored in WM (for sufficiently short songs) as a primacy gradient. When the top-down learned expectation pathway of the most active Phrase Chunk are activated by a rehearsal wave R (recall Figures 10A,B), it reads out Word Chunks that are stored in WM as a primacy gradient of the words in that phrase. Similarly, when the top-down learned expectation pathway of the most active Word Chunk is activated by a rehearsal wave R, it reads out the Item Chunks which constitute that word, again in a primacy gradient. Each Item Chunk, in turn, can read out the distributed pattern of features that it codes.

Each rehearsal wave node R is quickly inhibited by activation of the processing level whose chunk it activates. These inhibitory signals are activated by inhibitory gain control signals from the red disks that alternate with the blue rehearsal wave sources R.

For example, activating a Phrase Chunk sends excitatory signals to its Word Chunks. The most active Word Chunk is chosen by the circuit in Figures 10A,B. When that Word Chunk also receives a rehearsal wave from the rehearsal node R at its level, it can fire. In addition to sending top-down excitatory priming signals to the Item Chunk level, it also activates the inhibitory gain control node at its level, which inhibits the rehearsal node R at the Phrase Chunk level and thus terminates read-out of the next-most-active Phrase Chunk. This process of brief activation occurs at each level, terminated by an inhibitory gain control signal at the level just below.

When all the Items of an Item Chunk are rehearsed, the Item level can no longer activate an inhibitory gain control signal. Then a rehearsal wave can activate the currently most active Item Chunk, whose Items can be rehearsed in the same way. This process continues until all the Item Chunks of a Word Chunk are rehearsed. Then the next Word Chunk can be read out, and the process repeats itself until all the Word Chunks of a Phrase Chunk are rehearsed. And so on up the hierarchy until all the lyrics are performed.

The volitional rehearsal processes V and R in Figure 10 are regulated by the basal ganglia, whose substantia nigra pars reticulata (SNr) opens gates that release perceptual, cognitive, and motor processes (Hikosaka and Wurtz, 1983, 1989; Alexander and Crutcher, 1990; Mink and Thach, 1993; Alexander et al., 1996; Mink, 1996; Grahn and Brett, 2007, 2009; Grahn and Rowe, 2009, 2013; Chapin et al., 2010; Fujioka et al., 2010; Kung et al., 2013; Grossberg, 2016). Activating R is controlled by the basal ganglia *direct pathway*, whereas inhibiting R is controlled by the basal ganglia *indirect pathway*. Brown et al. (1999, 2004) describe a detailed neural model of basal ganglia circuitry that explains and simulates anatomical and neurobiological data about how this part of the brain works.

The polyvalent cells that fire in response to converging specific and nonspecific inputs have been a design feature in brains for many thousands, if not millions, of years, ranging from command cells in invertebrates to cells that regulate reinforcement learning, motivated attention, and decision-making in vertebrates. See Grossberg (1970, 1971, 1972a,b, 1974, 1978b, 2021) for neural models of such circuits. Whether the nonspecific input is excitatory, as in the simplified circuits of Figure 10, or an inhibitory signal whose activation disinhibits a tonically closed gating signal, as occurs throughout the basal ganglia (Alexander and Crutcher, 1990; Alexander et al., 1996), is a detail that will not be further discussed here.

### 4.4. Auditory Streaming, Speaker Normalization, and Learning What Lyrics Mean

Before discussing how lyrics can be learned and performed at different rhythms and beats, I will further develop the topic that was begun in Sections 1.3 and 1.7 of auditory streaming and speaker normalization in order to clarify how lyrics, or indeed any auditory communications, including language, can be learned by listening to a speaker whose sounds are uttered with different acoustic frequencies than our own voice produces.

Speaker normalization enables us to understand speech spoken by children, women, and men in different frequency ranges than our own. In particular, when babies babble sounds, they hear their own babbled sounds and use them to learn an associative map between their heard sounds and the motor commands that generated them. This kind of interaction is called a *circular reaction* by Piaget (1963). Babies use this associative map to learn how to imitate adult language sounds and thus to begin learning language.

Speaker normalization makes this possible. Without speaker normalization, the sounds that are produced by women and men could not activate the associative map that a baby learns, since it would have been learned between the sound frequencies that a baby can babble and the motor commands that made them. By first normalizing the sounds that the baby babbles, the associative map is learned between normalized sounds and these motor commands. Then sounds heard in different frequency ranges from women and men are also normalized, so that they can also activate the associative map to enable the baby to begin to imitate and learn the language utterances of other individuals. These language utterances include the lyrics of songs.

Before a baby or adult can process the sounds from an acoustic source, such as a speaker or singer, auditory streaming must first occur. Auditory streaming is also called *auditory scene analysis* (Bregman, 1990). It enables the sounds from multiple acoustic sources, including both voices and instruments, to be separated and tracked through time, including through intervals of noise or overlapping frequencies from multiple acoustic sources. Only after acoustic sources like voices are separated can they be individually normalized.

**FIGURE 11:**
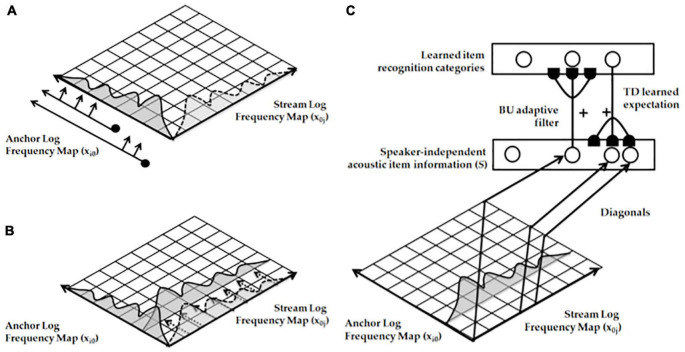
The NormNet model (Ames and Grossberg, 2008) shows how speaker normalization can be achieved using specializations of the same mechanisms that create auditory streams. See the text for details.

The above facts raise the question: How can brain evolution be smart enough to discover auditory streaming and the speaker normalization that follows it? Remarkably, both auditory streaming and speaker normalization seem to use homologous neural circuits. The ARTSTREAM model of auditory streaming (Figure 2; Grossberg et al., 2004) simulates how auditory streams are separated using strip maps, asymmetric competitive circuits, and ART category learning circuits. The circuits within the NormNet model of speaker normalization (Figure 11; Ames and Grossberg, 2008) replicate, in specialized form, the auditory streaming circuits whose outputs they process. NormNet hereby clarifies how auditory streaming supports speech normalization.

In particular, NormNet uses strip maps (Figure 11A) to simulate the transformation from speaker-dependent to speaker-normalized language representations (Figure 11B). After speech is transformed to become speaker-invariant (that is, normalized) and rate-invariant, it is in a form where our brains can learn and recognize language *meanings* from multiple speakers—without having to relearn them for each speaker and speech rate. These speaker-normalized spectral representations can be encoded through learning by speech item categories using Adaptive Resonance Theory circuits, which can quickly learn and stably remember them using both bottom-up and top-down adaptive pathways (Figure 11C). These speech item categories then input to an IOR working memory (Figure 3) whose primacy gradients can be categorized into syllable, word, and sentence list chunks.

NormNet was tested by simulating synthesized steady-state vowels from the Peterson and Barney (1952) vowel database and achieved accuracy rates similar to those achieved by human listeners. Vowels are, of course, the speech sounds that are most similar to musical pitches.

### 4.5. Changing Musical Key: Speaker Normalization and Relative Pitch

A basic issue in music is how to play or sing the same piece of music in a different key. I propose that a striking homology exists between normalizing speech and changing musical key. Just as speaker normalization uses a variant of the circuit that controls auditory streaming, changing a musical key uses a variant of the circuit that controls speaker normalization. Both processes shift all frequencies by a given amount, whether for purposes of speaking language or performing music. In Figure 11, this frequency is called the *anchor frequency*. Moreover, auditory streaming, speaker normalization, and changing musical key all use strip maps.

In particular, by normalizing speech, one can recognize a language utterance independent of its absolute pitches. By changing musical key, one can recognize a melodic utterance independent of its absolute pitches. In both processes, one can still hear the speech or music frequencies as they are uttered, while also recognizing their invariant meaning via parallel processing streams (Figure 3). Speaker normalization and changing musical key thus seem to use homologous circuits that each specialize mechanisms of auditory streaming. This homologous mechanism also makes it possible for the meanings of words sung in a different key to be understood.

### 4.6. Rehearsing a Phrase of a Melody’s Pitches at the Correct Rhythm

Having summarized how the *order information* of both lyrics and pitches may be stored in their respective working memories, the next issue is how they are performed at a desired rhythm. This issue will be discussed for the case of lyrics, leading to the ability to perform the words of a song at a learned rhythm. The same kind of analysis applies to the pitches with which the song is sung.

As noted in Section 1.3, a phrase of lyrics or melody can be stored in working memory and performed with a rhythm whose variable delays can be regulated by counting (Figure 8) the number of beats between notes, while inhibiting the performance of the next word or note for the correct duration until the count for the last word or note is complete.

Figure 8 summarizes part of the circuit in the parietal cortex that controls counting. The production of counts, whether vocally or unvocally, requires that this circuit interacts with prefrontal cortical circuits that regulate sequential performance of any list of items (Section 3) and with motor cortical circuits that read out the counting behaviors. As noted in Section 4.3, all of these processes are regulated by the substantia nigra pars reticulata (SNr) of the basal ganglia, which opens gates that release perceptual, cognitive, and motor processes.

The SNr also prevents the performance of behaviors whose gates are not open while counting is occurring. This happens using competitive interactions among the gating pathways. In particular, read-out of the next word in a lyric is prevented while counting the number of beats during which the current word is being performed.

### 4.7. Adaptively Timed Classical Conditioning: Cerebellar Spectral Timing

How are temporally discrete counts converted into a learned duration that is continuously read out during skilled performance while a given word is being performed? This process is controlled by adaptively timed learning within the cerebellum, which is known to be important in learning adaptively timed behaviors that include musical performance (Thach, 1998; Ramnani and Passingham, 2001; Sakai et al., 2002; Doyon et al., 2003; Ivry, 1997; Ivry et al., 2003; Penhune and Doyon, 2005; Ramnani, 2006; Zatorre et al., 2007).

**FIGURE 12:**
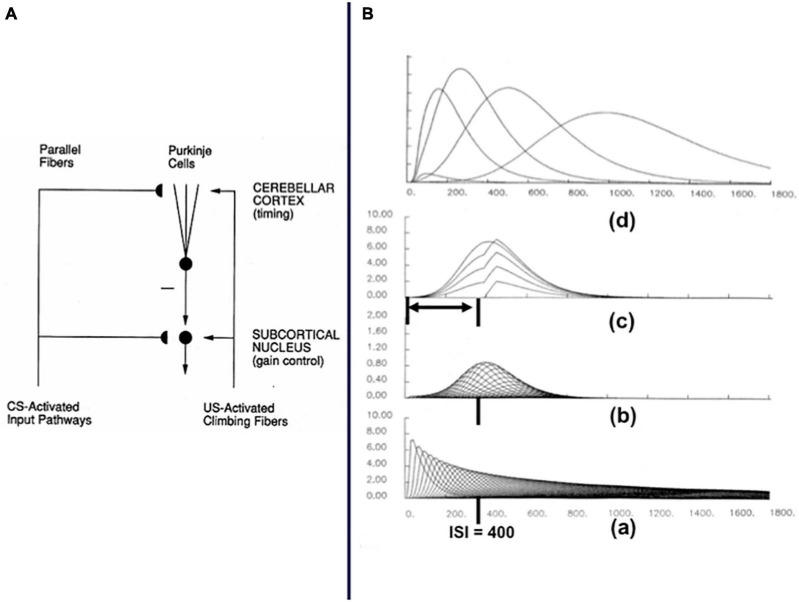
**(A)** Schematic of how learning in the cerebellum occurs. [Reprinted from Grossberg and Merrill (1996)]. **(B)** Summary of a computer simulation showing how the cerebellum learns adaptively timed responses over a duration of hundreds of milliseconds using the population response of a tunable spectrum of cells whose individual peak responses occur at different times during this time interval. [Adapted from Grossberg and Schmajuk (1989)]. See the text for details.

Adaptively timed learning by the cerebellum builds upon the Spectral Timing model, wherein a spectrum of cells that each react at different rates can together, as a population, learn to adaptively time movements that occur over a time span of hundreds of milliseconds or several seconds (Figure 12). Spectral timing circuits have also modeled adaptively timed learning by the hippocampus (Grossberg and Schmajuk, 1989; Grossberg and Merrill, 1992, 1996) and the basal ganglia (Brown et al., 1999, 2004), which contribute to dance movements in different ways, the former by regulating timed movements through space, and the latter by regulating when perceptual, cognitive, and movement gates open and close. The model was progressively developed until it could quantitatively simulate experimental facts about the biochemical processes that span such long times, notably the metabotropic glutamate receptor, or mGluR, system, with the spectrum specified by a Calcium gradient. Fiala et al. (1996) used this mGluR model to quantitatively simulate behavioral, neuroanatomical, neurophysiological, biophysical, and biochemical data about adaptively timed learning by the cerebellum. It has also been shown that the spectral timing circuits in basal ganglia, cerebellum, and hippocampus all share variations of the same circuit design.

Figure 12A summarizes how a learned sensory or cognitive representation sends trainable signals to both the cerebellar cortex and subcortical nuclei. In this figure, the sensory representation selectively responds to a conditioned stimulus, or CS, during a classical conditioning experiment. Classical conditioning is an ancient kind of learning that occurs across multiple species, ranging from invertebrates like *Aplysia californica* through rabbits to humans (Kamin, 1968; Smith, 1968; Millenson et al., 1977; Carew et al., 1981; Buonomano et al., 1990; Buonomano and Mauk, 1994; Woodruff-Pak et al., 1996). During classical conditioning, the CS starts out as a signal that may activate no learned associations. If the CS is paired on sufficiently many trials with an unconditioned stimulus, or US, which occurs after a delay of from 50 to several hundred milliseconds after the CS, then the CS can learn to activate some of the consequences that the US originally caused. During classical conditioning, the US acts like a positive or negative reinforcer, such as food or shock.

The trainable CS-activated signals to the cerebellar cortex are carried by parallel fibers that end in Purkinje cells (Eccles et al., 1967; Fiala et al., 1996). Learning that occurs at the parallel fiber/Purkinje cell synapses is called Long Term Depression, or LTD, because pairing at Purkinje cells of a CS-activated signal along a parallel fiber with a teaching signal along a US-activated climbing fiber causes the adaptive weight at the end of the active parallel fiber to decrease, or be depressed (Ito and Kano, 1982). Because Purkinje cells are tonically active, a smaller learned signal from a parallel fiber will activate target Purkinje cells less. These Purkinje cells will consequently inhibit their target subcortical nuclear cells less, thereby disinhibiting them and releasing a timed output signal downstream via the subcortical nucleus to which the trained Purkinje cells project.

Figure 12B provides a more detailed insight into properties of adaptively timed learning at the Purkinje cell synapses. I have called this kind of learning *spectrally timed* learning, or *spectral timing* for short (Fiala et al., 1996; Grossberg and Merrill, 1996), because a signal down the parallel fiber has multiple branches that activate a spectrum of cells, each of which reacts at a different rate (Figure 12Ba). Learning occurs at the synapses of each cell’s output pathway. More learning occurs in cells that are more active at times when the CS and US signals are paired. In the computer simulation results of Figure 12B, the most learning occurs at the interstimulus interval, or ISI, between the CS and US, which is 400 milliseconds. These learned weights multiply their signals in the spectrum, leading to a spectrum of learned signals that are largest at the ISI (Figure 12Bb). When all of these signals are added up at a fixed ISI, unimodal output signals are created which peak at the ISI, such as those shown in Figure 12Bc during the first five learning trials.

When the ISI is varied, the learning curves at multiple ISIs look like the curves in Figure 12Bd. These curves become broader as the ISI increases. This property is called the Weber Law (Smith, 1968). Note also that the envelope of all the curves has an inverted-U shape. The Weber Law is a signature of spectral timing wherever it occurs.

### 4.8. Learning to Perform Timed Lyrics Using Cerebellar Spectral Timing

**FIGURE 13:**
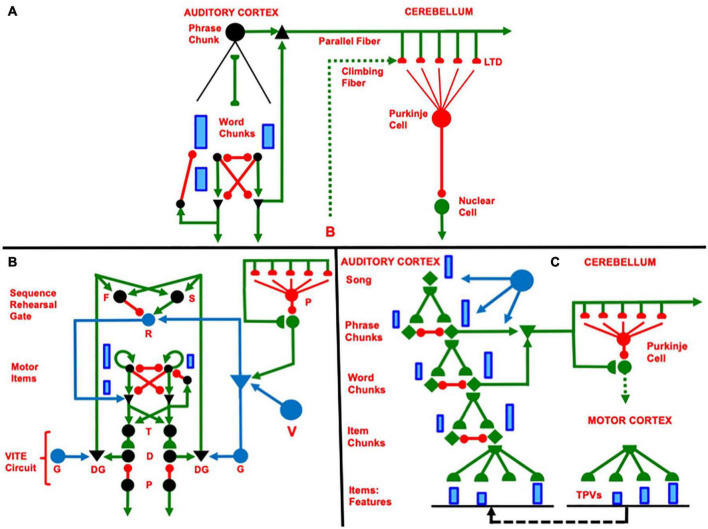
**(A)** Sequences of Word Chunks that are stored in working memory in the auditory cortex interact via adaptive bottom-up and top-down pathways in an ART circuit to learn Phrase Chunks. Each Phrase Chunk emits parallel pathways to polyvalent cells. Each polyvalent cell also receives a specific input from a particular Word Chunk. This polyvalent cell thus responds selectively to its Word Chunk when it is currently being performed as part of a prescribed phrase of lyrics. The polyvalent cell activates parallel fibers to the cerebellum where they learn an adaptively timed duration to perform that word in a lyric. Climbing fibers are teaching signals that drive adaptively timed learning using Long Term Depression, or LTD, in (parallel fiber)—(Purkinje cell) synapses. During such a trained duration, Purkinje cell outputs are depressed and their target subcortical nuclear cells are disinhibited. **(B)** Circuitry whereby cerebellar adaptive timing regulates the learned durations between successive words in a lyric. **(C)** Circuitry whereby cerebellar adaptive timing regulates timed performance by the hierarchy of processing stages in the auditory cortex that represent a song’s lyrics. See the text for details.

When counting signals that occur regularly in time activate training inputs via climbing fibers to a cerebellar spectrum then, as Figure 12 shows, the Purkinje cell output will learn to remain small throughout that time interval. In this way, training inputs that occur discretely in time are converted into a learned spectrum that remains small throughout a timed duration during which it disinhibits its target cerebellar nuclear cells (Figure 13A).

How is the correctly learned timed duration linked to a given word in a song’s lyrics? This requires an interaction between multiple brain regions, including the thalamus, cerebral cortex, cerebellum, and basal ganglia (Grossberg and Paine, 2000; Bostan and Strick, 2010; Bostan et al., 2013; Gao et al., 2018; Hintzen et al., 2018). Figure 13A shows how a Phrase Chunk, which can code for a learned word, can accomplish this. As in Figure 13C, such a Phrase Chunk can read out its series of words into an IOR working memory. It can also, in parallel, read out an array of pathways that activate a band of parallel fibers into the cerebellum (Figure 13A). Before reaching the cerebellum, each pathway inputs to a polyvalent cell that also receives an excitatory input from a Word Chunk in working memory when it generates an output signal in order to be rehearsed. Convergent signals from the Phrase Chunk and the Word Chunk at this polyvalent cell can fire it, so that its output signal to the cerebellum only samples counting inputs when a particular word in a prescribed phrase of lyrics in a song is being rehearsed. In this way, for example, when the word “true” in the phrase “my true love is true” (Section 1.7) is read out of working memory, it can learn to be performed with two different durations at its two locations in the phrase.

Figure 13B shows that the disinhibited cerebellar nuclear cell inputs to a polyvalent cell (triangle in blue) which also receives a volitional signal V from the basal ganglia. When both inputs are on, the polyvalent cell can fire and support rehearsal of the word using the LIST PARSE circuit in Figure 6. LIST PARSE can control timed read out of a word at variable speeds by using acceleration and deceleration signals to control a smooth transition to the next sound in the word as performance of the previous sound is almost completed by a Vector Integration to Endpoint, or VITE, motor trajectory controller. This happens in the following way.

This Vector Integration to Endpoint, or VITE, model and its extensions (e.g., Bullock and Grossberg, 1988, 1991; Bullock et al., 1993) were originally used to explain and quantitatively simulate psychophysical and neurobiological data about arm movement reaches. VITE dynamics have also been shown to play a role in controlling other types of movements, including movements to play musical instruments, and movements of the speech articulators that are used to sing (Guenther, 1995; Jacobs and Bullock, 1998; Bohland et al., 2010).

The individual movements that are controlled by LIST PARSE in Figure 6 (right panel) use a VITE model circuit. In such a circuit, a *present position vector* P_i_ computes the current position of a limb, and a *target position vector* T_i_ computes the desired final position of the limb. P_i_ is subtracted from T_i_ (dashed red line between them) to compute a *difference vector* D_i_ that codes the direction and distance of the desired straight movement. Then D_i_ is multiplied by a volitional GO signal G to determine an *outflow movement speed vector* D_i_G that is integrated through time by P_i_ until P_i_ equals T_i_, at which time the desired target position has been reached and the difference vector equals zero. Increasing G increases movement speed.

LIST PARSE can interact with a VITE trajectory generator to smoothly perform a sequence of straight movements at variable speeds, such as a skilled sequence of arm movements, dance, or sounds in a word. Given that this can be achieved at variable speeds by varying the size of a volitional signal V, how do the circuits in Figure 6 know when an ongoing straight movement is almost complete, so that the next straight movement can begin to be rehearsed from working memory in a smooth way?

In Figure 6, the rehearsal signal R that controls read-out of successive movement commands from motor working memory is regulated by a signal (B – A) that is activated by cells that slowly (B) and more quickly (A) time-average outflow velocity signals DG. Due to the bell shape of the velocity vector DG for each straight movement, (B – A) is sensitive to whether DG is increasing, and thus accelerating in its growth, or decreasing, and thus decelerating. Because of the bell shape of DG, (B – A) is initially negative, thereby keeping R off, but (B – A) becomes positive toward the end of the movement, thereby initiating a smoothly interpolated rehearsal of the next movement in the performance of the word. As schematized in Figure 13C, then the next word in the phrase can be rehearsed with its learned timing until the entire musical phrase has been performed with the rhythm of the song.

The rate at which such movement trajectories may be completed in response to a GO signal G of a given size helps to determine the “resonance” frequency of performing a sequence of such movements. This kind of circuit may clarify how preferred performance rates during walking and running are linked to rates of musical performance. Various studies have suggested that “people can synchronize their walking movements with music over a broad range of tempi, but that this synchronization is most optimal in a rather narrow range around 120 BPM [beats per minute]. This finding can be connected with previous findings indicating that most music has a tempo in this range” (Styns et al., 2007, p. 784).

## 5. Basal Ganglia Control of Periodic Dynamics, Including Beats

A great deal has been written about the psychology and neurobiology of musical beat in both normal, or typical, individuals and clinical patients, including how it engages sensory, cognitive, emotional, and motor systems in both musicians and non-musicians (e.g., Jones, 1976; London, 2004; Buhmann et al., 2017; Rajendran et al., 2017; Tal et al., 2017; Beier and Ferreira, 2018; Lenc et al., 2018; Slater et al., 2018; Lagrois et al., 2019; Toiviainen et al., 2019), and models have been proposed to clarify the underlying mechanisms (e.g., Lerdahl and Jackendoff, 1983; Large, 2010; Large et al., 2015). It has also been shown that interactions exist across rhythmic circuits that oscillate with very different frequencies, such as circadian and motor circuits (Ivanov et al., 2007).

I will not review this extensive literature here. Rather, I will briefly note how the same kind of recurrent shunting on-center off-surround network that has above been used to explain and simulate multiple types of data can also create beat-like oscillations whose frequency can be regulated by volitional signals.

### 5.1. Finger and Gait Oscillations: Shunting Recurrent Competition With Slow Inhibition

**FIGURE 14:**
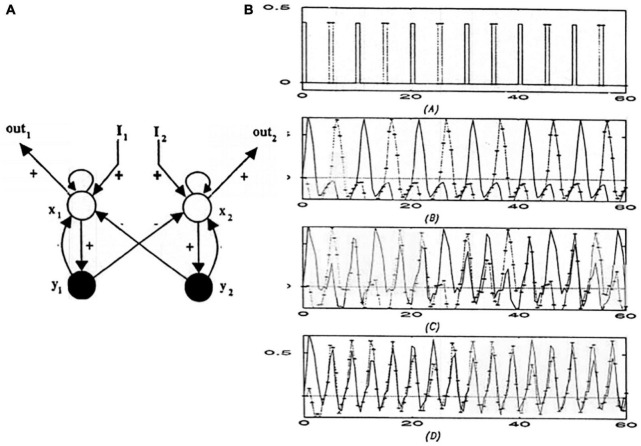
A recurrent shunting on-center off-surround network models data about synchronization of bimanual tapping. Bifurcation from anti-phase to in-phase oscillation occurs in response to anti-phase inputs of increasing frequency. See the text for details. [Reprinted from Grossberg et al. (1997b)].

The circuit in Figure 14A is defined by a recurrent shunting on-center off-surround circuit (Grossberg et al., 1997b). It can oscillate in response to external volitional inputs I_1_ and I_2_ because the inhibitory interneurons in its off-surround vary more slowly through time than the self-excitatory cells in the on-center. As a result, the recurrent on-center can rapidly amplify the activity of its cell, followed by slow inhibition that shuts it down, after which another cell can get activated.

This kind of oscillator has been used to simulate oscillatory movements of fingers and legs. Concerning finger movements, Yamanishi et al. (1980) described a bimanual finger tapping task whereby subjects were required to start from a stable posture before periodically tapping keys in time to visual cues that occur at an increasing frequency (Figure 14B). When one of the periodic inputs I_1_ or I_2_ is missing, then the recurrent interactions continue to oscillate, as in the missing beat phenomenon (Tal et al., 2017).

**FIGURE 15:**
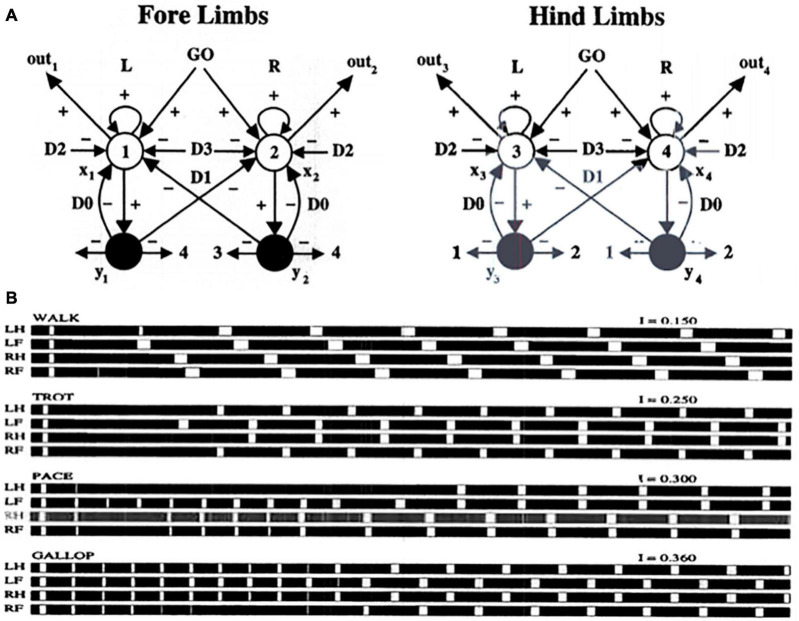
A pair of interacting recurrent shunting on-center (+ signs) off-surround (– signs) networks model the central pattern generator, or CPG, that controls quadruped movement gaits. Movement gait transitions are activated by an increasing volitional GO signal. Cells that emit excitatory signals are denoted by open circles. Inhibitory interneurons that emit inhibitory signals are denoted by closed disks. **(A)** Self-inhibitory feedback is labeled by the parameter D0, inhibition between forelimbs and between hindlimbs is labeled by D1, inhibition between matched forelimbs and hindlimbs is labeled by D2, and inhibition between crossed forelimbs and hindlimbs is labeled by D3. **(B)** Computer simulation of how increasing the GO signal, when it is combined with GO signal modulation of the inhibitory coefficients, triggers an ordered series of gaits (walk, trot, pace, and gallop). [Reprinted with permission from Pribe et al. (1997)].

Figure 15A describes a recurrent shunting on-center off-surround network for control of quadruped locomotion from the spinal cord (Pribe et al., 1997). Here, opening an appropriate gate in the SNr disinhibits a GO signal. Increasing the GO signal causes the circuit to generate gaits and gait transitions that are familiar in quadrupeds like cats (walk-trot-pace-gallop; Figure 15B), humans (walk-run), and elephants (amble-walk). In the case of music, such a circuit can control a beat that can be sped up or slowed down under volitional control.

### 5.2. A Similar Design for Beat and Movement Circuits Explains How They Synchronize

Recurrent shunting on-center off-surround circuits, such as those in Figures 14, 15, may be used to generate musical beats, while also clarifying how synchronization of musical beat circuits can occur with similarly designed motor circuits that enable us to move with the beat (Wallin et al., 2000; Phillips-Silver and Trainor, 2007; Trainor, 2007; Zatorre et al., 2007; Sowiński and Bella, 2013; Burger et al., 2014; Schaefer and Overy, 2015). The observation that music can induce movement goes back at least to Aristotle who asserted that movement “follows” sound (von Helmholtz, 1954; Phillips-Silver and Trainor, 2007).

## 6. Concluding Remarks

Two of the main insights of this article are the following: First, all the brain circuits that process lyrics, melodies, rhythms, and beats are specializations of a shared neural design; namely, recurrent shunting on-center off-surround networks. Second, these various networks arose early during human evolution to achieve more basic survival demands.

Periodic oscillations are used by our brains to support multiple rhythmic activities. The same types of oscillatory circuits that control periodic motor behaviors, such as walking and running, are also proposed to support musical beats, thereby clarifying the strong urge to move with the beat.

Regular rhythms can be learned using cortical modulation of beats that are generated in the basal ganglia. Arbitrary rhythms, whether regular or irregular, can be learned when cerebellar adaptively timed learning circuits interact with cortical and basal ganglia mechanisms. The type of spectral timing found in the cerebellum also controls many kinds of adaptively timed brain processes, ranging from classical conditioning in invertebrates to musical performance by humans.

Emotional, cognitive-emotional, and expectation violation processes in music are not analyzed in this article, but neural models to help do this in future studies are summarized in the Supplementary Information.

## Author Contributions

The author confirms being the sole contributor of this work and has approved it for publication.

## Conflict of Interest

The author declares that the research was conducted in the absence of any commercial or financial relationships that could be construed as a potential conflict of interest.

## Publisher’s Note

All claims expressed in this article are solely those of the authors and do not necessarily represent those of their affiliated organizations, or those of the publisher, the editors and the reviewers. Any product that may be evaluated in this article, or claim that may be made by its manufacturer, is not guaranteed or endorsed by the publisher.

## Footnotes

1
https://www.youtube.com/watch?v=dpGYmYC3p0I

2
https://www.youtube.com/watch?v=57tK6aQS_H0
