# Supplementary material for: Toward Understanding the Brain Dynamics of Music: Learning and Conscious Performance of Lyrics and Melodies With Variable Rhythms and Beats
Source: Front Syst Neurosci. 2022 Apr 8;16:766239. doi: 10.3389/fnsys.2022.766239 (PMC9028030; doi:10.3389/fnsys.2022.766239)
Supplement: Supplementary file 1 [file Data_Sheet_1.docx]

**SUPPLEMENTARY INFORMATION**

**Oscillators to carry a beat**

***Ellias-Grossberg oscillator.*** Perhaps the simplest shunting competitive network that can be used as a central pattern generator, or CPG, is the Ellias-Grossberg model (Ellias and Grossberg, 1975). Here, a single cell excites itself (*x*) and sends excitatory signals to, and receives inhibitory feedback from, a slow inhibitory interneuron (*y*):

$dx/dt=-Ax+(B-x)[f(x)+I]-Dxf(y)$ (1)

$dy/dt=E(x-y).$ (2)

Ellias and Grossberg (1975) prove theorems that characterize how this system oscillates when the GO input I in (1) is sufficiently large. The oscillation frequency then increases with I until it reaches a critical value, after which it decreases.

To quantitatively explain data about oscillatory movements, equations (1) and (2) were generalized in three ways: In (1), inhibition drives the excitatory potential to 0, which is also the passive equilibrium point. More generally, large inhibitory inputs *hyperpolarize* a cell. Also in (1), the positive feedback signal $f(x)$ uses the same signal function as the inhibitory feedback signal $f(y)$. In general, this is not true. Finally, in (2), there is no shunting inhibitory term. The simplest system that includes these three properties is:

$dx/dt=-Ax+(B-x)[f(x)+I]-(C+x)Dg(y)$ (3)

$dy/dt=E[(1-y)[x]^{+}-y].$ (4)

In equation (3), the term $(C+x)$ allows $x$ to become hyperpolarized to the value $-C,$ and the term $g(y)$ defines an inhibitory feedback signal that may differ from the excitatory feedback signal *f(y).* In equation (4), the shunting term $(1-y)$ defines a maximal possible value of 1 for$y$, and the threshold function $[x]^{+}=\max(x,0)$ equals $x$ when $x$ is non-negative, and $0$ when $x$ is negative.

This system can be generalized to the following system of shunting recurrent competitive equations which, in different specialized anatomies, quantitatively simulate oscillations that occur when humans coordinate finger movements across both hands (Figure 14; Grossberg, Pribe, and Cohen, 1997) and quadruped animals move with one of several possible gaits (Figure 15; Pribe, Grossberg, and Cohen, 1997):

$dx_{i}/dt=-Ax_{i}+(B-x_{i})[f(x_{i})+I_{i}]-(C+x_{i})\sum_{j} D_{ij}g(y_{j})$ (5)

$dy_{i}/dt=E[(1-y_{i})[x_{i}]^{+}-y_{i}].$ (6)

***Finger oscillations: From anti-phase to in-phase synchronization and missing beat.*** Yamanishi, Kawato, and Suzuki (1980) described a bimanual finger tapping task whereby subjects were required to tap keys in time to visual cues. The timing of the cues was varied across ten relative phases. Subjects’ fingers tended to slip from intermediate phase relationships towards being in-phase or anti-phase. The in-phase and anti-phase relationships exhibited less variability than intermediate phase relationships, leading to a “seagull” effect to describe the standard deviation of observed movements as a function of relative phase. Yamanishi, Kawato, and Suzuki (1980) also described a formal phase oscillator to describe these effects. Grossberg, Pribe, and Cohen (1997) showed how these data properties emerge from system (5) and (6) when the inputs $I_{i}$ copy the timing of the experimental inputs. Moreover, if one of the inputs is omitted, then the recurrent interactions will continue to oscillate, thereby exhibiting the missing pulse phenomenon (Tal et al., 2017).

Kelso (1981) described a related task in which a metronome signaled when the fingers should move. Both in-phase and anti-phase movements were possible at lower frequencies, but the movements spontaneously switched to in-phase movements at higher frequencies (Kelso, 1984). These properties were also simulated by equations (5) and (6). Figure 14a describes the CPG oscillator that couples the two fingers when i = 1, 2. Figure 14b shows a computer simulation of CPG dynamics wherein anti-phase inputs in (A) to equation (5) first lead to anti-phase finger movements, as simulated in (B), that spontaneously switch to in-phase movements as the anti-phase inputs occur more rapidly, as simulated in in (C) and (D).

These results do not incorporate influences of perceptual, cognitive, and motor feedback factors that include different delays in processing visual or auditory periodic stimuli, different delays of sensory and motor afferent pathways, temporal dynamics of motor cortical movement commands, dynamics and kinematics of opponent motor effectors, effects of afferent feedback from movements on their controllers, anticipatory properties of prefrontal cortical circuits, and so on. Bruno Repp has particularly well documented such subtleties during finger movement synchronization (e.g., Repp, 2005, 2006a, 2006b; Repp and Su, 2013). Such additional influences may help to explain why musicians typically do not keep time to a strict periodic beat (Hennig et al., 2011). Equations (5) and (6) model a core oscillator that these various inputs can modulate in future studies.

***Quadruped gait oscillations and performance of motor sequences at variable speeds.*** A main difference between finger synchronization studies and quadruped gaits is that, during finger synchronization studies, external inputs drive finger synchronization (Figure 14), whereas during quadruped gaits, an internal GO signal operates (Figure 15a) whose increasing size triggers different gaits as the legs oscillate faster (Figure 15b). Quadruped movements also need to cope with the force of gravity to maintain bodily balance despite movement-generated forces through time (Brown, 1911). One important challenge is ensuring that one leg sufficiently stabilizes the body while the other leg is being launched towards the body’s next target position, and to do so at variable speeds. How does one foot know how far along the other foot has gone, and to do it at variable speeds? The circuit in Figures 6 and 13b clarifies how may happen.

**Models to help explain emotion, cognitive-emotional, and expectation violations in music**

This article will not try to explain how emotions may motivate and be engaged by music. I am postponing this important topic to a future article despite the fact that my colleagues and I have also been incrementally developing a comprehensive neural theory of emotion and cognitive-emotional interactions for over 40 years (e.g., Dranias, Grossberg, and Bullock, 2008; Fiala, Grossberg, and Bullock, 1996; Franklin and Grossberg, 2017; Grossberg, 1971, 1972a, 1972b, 1975, 1982, 1984, 2017a, 2017c, 2018, 2019, 2021; Grossberg, Bullock, and Dranias, 2008; Grossberg and Levine, 1987; Grossberg and Merrill, 1992, 1996; Grossberg and Schmajuk, 1987, 1989)). This omission is needed because the topics that the article does discuss are so substantial.

In anticipation of a future analysis, let me note here that the results of our cognitive-emotional model, whose variations are called CogEM (Cognitive-Emotional-Motor) and MOTIVATOR (Matching Objects To Internal VAlues Triggers Option Revaluations), may be used to clarify emotional reactions to musical experiences, much as they have been used to explain emotional reactions to perceptual and cognitive experiences, including how motivated attention helps to pay sustained attention to emotionally valued information and thereby control temporally coordinated actions to realize valued goals. In the case of music, such sustained attention helps to sustain performance of a piece of music, modulated by the emotions felt through time by the performer.

Available neural models also clarify how disconfirmation of expectations in a piece of music may influence emotional reactions to it (e.g., Abdallah and Plumbley, 2009; Egermann et al., 2013; Hanslick, 1854). Indeed, the cognitive and neural theory called Adaptive Resonance Theory, or ART, that will play a central role in the subsequent discussions, clarifies how learned expectations dynamically stabilize all kinds of cognitive and emotional learning (Figures 3 and 4; Grossberg, 1980b, 2013a, 2017c), including how music may be learned rapidly without incurring the risk of catastrophically forgetting musical phrases that have already been learned. Disconfirmation of these expectations, whether during musical or non-musical experiences, leads to widespread cognitive and emotional events, including antagonistic rebounds leading to reset of currently active emotions, that enable us to adapt to changing environmental events, whether in the forest primeval or the concert hall.
